# Most self-touches are with the nondominant hand

**DOI:** 10.1038/s41598-020-67521-5

**Published:** 2020-06-26

**Authors:** Nan Zhang, Wei Jia, Peihua Wang, Marco-Felipe King, Pak-To Chan, Yuguo Li

**Affiliations:** 1 Department of Mechanical Engineering, The University of Hong Kong, Pokfulam Road, Hong Kong, People’s Republic of China; 2 Zhejiang Institute of Research and Innovation, The University of Hong Kong, Lin An, Zhejiang, People’s Republic of China; 3 School of Civil Engineering, The University of Leeds, Leeds, UK; 4 School of Public Health, The University of Hong Kong, 7 Sassoon Road, Pokfulam, Hong Kong, People’s Republic of China

**Keywords:** Infectious diseases, Microbiology

## Abstract

Self-touch may promote the transfer of microorganisms between body parts or surfaces to mucosa. In overt videography of a post-graduate office, students spent 9% of their time touching their own hair, face, neck, and shoulders (HFNS). These data were collected from 274,000 s of surveillance video in a Chinese graduate student office. The non-dominant hand contributed to 66.1% of HFNS-touches. Most importantly, mucous membranes were touched, on average, 34.3 (SE = 2.4) times per hour, which the non-dominant hand contributed to 240% more than the dominant hand. Gender had no significant effect on touch frequency, but a significant effect on duration per touch. The duration per touch on the HFNS was fitted with a log–log linear distribution. Touch behaviour analysis included surface combinations and a probability matrix for sequential touches of 20 sub-surfaces. These findings may partly explain the observed variation in the literature regarding the microbiome community distribution on human skin, supporting the importance of indirect contact transmission route in some respiratory disease transmission and providing data for risk analysis of infection spread and control.

## Introduction

It is known that some respiratory and enteric viruses, such as rhinovirus and norovirus may be transmitted by touching mucous membranes with our own contaminated hands^1–3^, and a vast number of bacteria thrive on human skin^4,5^. Skin is one of the largest human-associated microbial habitats and harbours up to $1\times {10}^{7}$ bacteria per cm^2^ of skin, which can have important effects on health^6,7^. Average skin bacterial communities appear to be more diverse than those found in the throat, stomach or faecal environments^8^. The hands play a critical role in microbiome transfer via frequent contact with contaminated environmental surfaces and a typical hand harbours more than 4,700 unique phylotypes^6^. Average phylotype richness on a single palm surface is also more than three times higher than the variety observed in molecular surveys of both forearm skin^5^ and elbow skin^9^.

The factors that drive this variability on the hand and skin bacterial community composition remain poorly understood^6,10^. Age, hand and gender are intrinsic factors that affect the composition of the hand microbiome^6,11^ due to the high variability in the population’s skin humidity, acidity and nutrient level, sweat or sebum production, frequency of moisturiser or cosmetic application and skin thickness^12,13^. It is known that touch can transfer microbes between hands and surfaces, but very little is known about human touch behaviour. A recent study showed that students touched surfaces with both hands for more than 90% of the observed time in their office, and more than 10% of touch time was on their own hair, face, neck and shoulders (HFNS)^14^. These body surfaces can be contaminated by our own contaminated hands. Therefore, the distribution of touches to these surfaces is thus important.

The importance of touch is also associated with the survival of microbes on the hands. Some enteric viruses such as norovirus can live for extended periods on human skin^15^. Whilst some respiratory viruses such as influenza A have a high inactivation rate on human hands, with a survival time of less than 10 min, it commonly leads to the conclusion that the fomite route is less important than the airborne or large droplet routes^16–18^. Most self-touches are directed towards the face and hands^19,20^. If the self-touching frequency of the nostrils or other HFNS parts is high, there is a risk that viable virus will be transferred to these surfaces before their death.

In this study, we analysed how students in a graduate student office touched their own HFNS from 9 a.m. to 9 p.m. on 5 successive weekdays between September 11th and 15th, 2017. Our experimental protocols were approved by Institute of Public Safety Research, Tsinghua University and adhere to guidelines and regulations of Nature Journals. All participated students signed an informed consent form before the experiment and all of them were over 18. All of the touch episodes were recorded by four video cameras installed on the office ceiling at carefully chosen locations. Data were collected by second-by-second inspection of the video footage by five trained video analysts. The collected information included the onset time, duration, the identity of the student, the hand used, and the surface touched.

## Methods

In a previous study, we found that the students in a Chinese graduate student office touched themselves an average of 98.7 times per hour and that more than 50% of these touches were on their own HFNS^14^. Here, the self-touch behaviour on these parts of the human body with a high touch frequency is analysed using these data.

### Room setting

The office measured 12.0 × 8.4 × 2.7 m. Thirty-nine students worked in the office, and each was assigned a code (from *P*_*0*_ to *P*_*38*_). All of the students were monitored by four video cameras from 9 a.m. to 9 p.m. on 5 successive weekdays (Monday to Friday). All video cameras were installed on the office ceiling. All of the students had been notified of video monitoring before the experiment. All of the students except students *P*_*2*_, *P*_*5*_, *P*_*6*_ and *P*_*36*_ were monitored by at least two cameras, which enabled us to obtain high-resolution (1080P) visual–spatial–temporal data. During the 5 days of recording, only 29 students (17 male) used the office. Students *P*_*6*_ and *P*_*12*_ are left-handed when they eat and right-handed for some routine activities in the office, such as writing and manipulation of an object. Therefore, these two students are still regarded as right-handed in this study. All of the students were between 21 and 29 years of age, except for one female student (37 years). All participants were registered as Master’s degree or PhD students at the time of the study.

### Surface introduction

**Figure 1:**
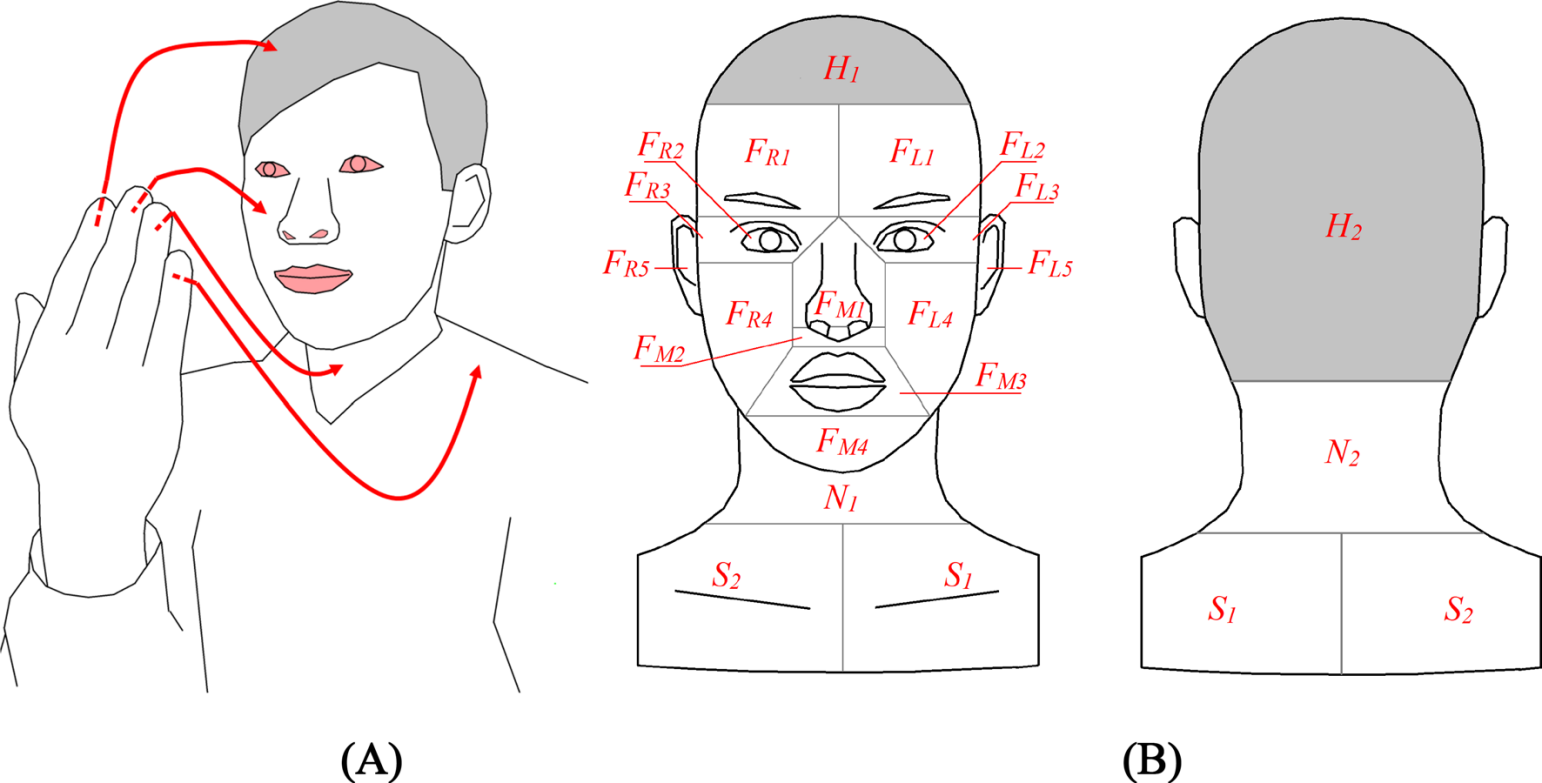
(**A**) Illustration of touch action on hair, face, neck and shoulders (HFNS), respectively, with mucous membranes shown in red; (**B**) 20 sub-surfaces with surface codes listed in Table 1.

We focused on self-touch behaviour on four surfaces: the HFNS (Fig. 1A). Table 1 shows the surface classification and codes for all of the surfaces. ‘Hair’ is divided into the front and back parts (*H*_*1*_ and *H*_*2*_). ‘Face’ is divided into left, middle, and right. Both left and right parts include the forehead, the eyes, the peripheral area of the eyes, the cheeks and the ears (*F*_*L1*_ to *F*_*L5*_; *F*_*R1*_ to *F*_*R5*_), and the middle includes the nose, nostrils, lips and chin (*F*_*M1*_ to *F*_*M4*_). ‘Neck’ includes the front and back parts (*N*_*1*_ and *N*_*2*_), and ‘shoulders’ includes the left and right parts (*S*_*1*_ and *S*_*2*_). Therefore, there were 20 sub-surfaces (Fig. 1B) in HFNS. The eyes (*F*_*L2*_ & *F*_*R2*_), nostrils (*F*_*M2*_) and lips (*F*_*M3*_), shown in red in Fig. 1A, are the mucous membranes on the face.

**Table 1:** Hair, face, neck and shoulders (HFNS) sub-surface classification and their code assignment.

Surface	Subsurface	Code	Surface	Subsurface	Code
Hair	Front top of hair	*H*_*1*_	Face (left)	Left forehead	*F*_*L1*_
	Back hair	*H*_*2*_		Left eye	*F*_*L2*_
Neck	Front neck	*N*_*1*_		Peripheric area of left eye	*F*_*L3*_
	Back neck	*N*_*2*_		Left cheek	*F*_*L4*_
Shoulders	Left shoulder	*S*_*1*_		Left ear	*F*_*L5*_
	Right shoulder	*S*_*2*_	Face (right)	Right forehead	*F*_*R1*_
Face (middle)	Nose (upper part)	*F*_*M1*_		Right eye	*F*_*R2*_
	Nostril	*F*_*M2*_		Peripheric area of right eye	*F*_*R3*_
	Lips	*F*_*M3*_		Right cheek	*F*_*R4*_
	Chin	*F*_*M4*_		Right ear	*F*_*R5*_

### Training and evaluation of video analysts

All video analysts were specially trained for this image analysis. The training included analysis of a sample test video and comparison of their estimated data with the real actions. The analysts were only allowed to begin the formal study data collection when their accuracy reached at least 95% on the sample test video.

In the 5-day video records, 3,035,916 s of touching data were collected. A total of 283,221 s of data were found to be related to touches of the HFNS, and 2.4% (6,789 of 283,221) were difficult to identify accurately because the view was blocked by people or objects. Of the remaining 276,342 s of touching data, 1,934 s were on another subject’s HFNS. Therefore, 274,408 s valid data for self-touches of HFNS remained. All of the results obtained in this study were from these valid data.

For quality control during video image analysis, one author (NZ) verified the close contact data collected in 1 of every 200 rows. Thus, of the 274,408 rows of data, 1,372 rows were checked for accuracy. (Each row has four elements: who touched the surface, which hand touched the surface, which surface was touched and what time the surface was touched.) Of these, 3.2% (173 of 5,448 elements) were found to be incorrectly recorded by the original video analysts and were corrected.

### Video data processing

During the data collection from all of the clips, the five video analysts recorded which surfaces were touched. In this study, a touch is defined as continuous contact between a hand (palm/finger/back of hand) and a sub-surface of the HFNS until the hand is detached from the surface for more than 1 s. For example, if a student frequently touched his/her nose and the interval of each touch was less than 1 s, only one touch was recorded. If a student touched his/her nose first and then moved the hand to his chin within an interval of 1 s, two sub-surface touches were recorded (i.e., the nose and the chin). However, only one touch was recorded for the face because both the chin and left cheek belong to the face, and the hand did not leave the face for more than 1 s. For data recording, video analysts collected all sub-surfaces each second. For example, a student touched his/her nostrils and lips at the same time, ‘*F*_*M2*_ & *F*_*M3*_’ was recorded. If a student scratched his/her head quickly between the front and back of the head, ‘*H*_*1*_ & *H*_*2*_’ was recorded. The total 274,408 s of valid data include all self-touches of each individual sub-surface, and each touch on two or more sub-surfaces was counted twice or more.

Automated video analysis could not be achieved satisfactorily and therefore manual observation was conducted. The video was analysed by five trained analysts appointed to process the data second-by-second, recording all visible touches related to the HFNS. The video was played at normal speed (1×) and was paused every second using PotPlayer 64 bit (https://potplayer.daum.net/). The five analysts typed the data into an Excel file (Table 2). The information recorded included the onset time, duration, the touched surface and the hand used. The four surfaces and 20 sub-surfaces are defined in Fig. 1.

**Table 2:** Data collection form for video analysts.

Date^a^	Time^b^	Student ID^c^	Hand^d^	Surface^e^
September 11 to 15	09:00:00–20:59:59	0 to 38	Left/right	Code

^a^Date: 5 consecutive days from September 11 to 15, 2017.

^b^Time: the recorded time from 09:00:00 to 20:59:59 with a resolution of 1 s.

^c^Student ID: the student who touched his/her hair, face, neck or shoulders (coded from *P*_*0*_ to *P*_*38*_).

^d^Hand: which hand the student used to touch his/her own HFNS.

^e^Surface: the code of touched sub-surfaces as shown in Fig. 1 and Table 1.

### Data analysis

Datasets including gender, hand, sub-surface, touch frequency and duration per touch were generated based on the above 274,408 s of valid data. Touch frequency and duration per touch were based on each subject, each consecutive day and hand. Touch frequency was analysed in a 3-way analysis of variance (ANOVA), with a between-subject factor of group (genders/hands/sub-surfaces), within-subject factors of hand (dominant hand/non-dominant hand), gender (male/female) and subsurface (20 sub-surfaces). Before testing, six hypothetical tests were carried out, e.g. outlier test, normality test using the Shapiro Wilks test and equality of variance using Levene’s test. Analysis on duration per touch was carried out using a 3-way ANOVA with the same hypothetical tests and conditions as described above. Post-hoc comparisons of interactions were performed using a one-way ANOVA. Pairwise comparison with Bonferroni correction was carried out to perform group difference and decrease Type I error.

The sub-surfaces were grouped into five parts, hair, non-mucosal face, mucous membranes, neck, and shoulder, respectively. Datasets are adjusted according to Eqs. (1) and (2):

1 $${TF}_{y}={\sum }_{x\in {X}_{y}}{TF}_{x}$$

2 $${DPT}_{y}=\frac{{\sum }_{x\in {X}_{y}}{T}_{x}}{{\sum }_{x\in {X}_{y}}{t}_{x}}$$

where ${TF}_{y}$ and ${TF}_{x}$ indicate touch frequency of surface y and sub-surface x, respectively; ${X}_{y}$ indicates set of all surfaces belonging to surface y; ${DPT}_{y}$ is duration per touch of surface y; ${T}_{x}$ is total touch duration of sub-surface x; ${t}_{x}$ is how many touches on sub-surface x. All above descriptions are based on the dimension of specific subject, hand and date.

Analyses on touch frequency and duration per touch by grouping surfaces were carried out using a 3-way ANOVA with the same hypothetical tests and conditions as described in the above sub-surface analysis. A one-way ANOVA and pairwise comparison were also performed to get comparisons of interactions between subjects and individual effects within subjects. Analysis on grouping sub-surface let us know how mucous membranes act in self-touch behaviours.

## Results

### Basic results of self-touch behaviours

The average touch frequency on the surfaces was 52.8 times per hour, and the average duration per touch (*t*_*d*_) was 12.3 s (Table 3). Of all of the touches, 24.0%, 76.9%, 6.2% and 2.6% were on the hair, face, neck and shoulders, respectively, which means that each student self-touched the hair, face, neck and shoulders 12.7, 40.6, 3.3 and 1.4 times per hour, respectively. The face had the highest touch frequency; 76.9% of touches and 85.3% of touch time on the HFNS was related to the face.

**Table 3:** Touch frequency on different combinations of surfaces.

Surface^a^	*H*	*F*	*N*	*S*	*HF*	*HN*	*HS*	*FN*
Frequency^b^	8.1	36.5	1.8	1.2	3.6	0.9	0	0.4
Duration^c^	8.0	14.5	7.3	8.4	6.7	6.9	–	13.9

Surface	*FS*	*NS*	*HFN*	*HFS*	*HNS*	*FNS*	*HFNS*	All
Frequency	0	0.1	0	0	0	0	0	52.8
Duration	–	11.2	–	–	–	–	–	12.3

^a^*H*, *F*, *N* and *S* indicate hair, face, neck and shoulders, respectively. For example, *HF* means the person touches his/her hair and face with one hand at the same time.

^b^Touch frequency (unit: time/h).

^c^Duration per touch (unit: s).

There were 22,271 valid self-touches of various combinations of surfaces. In all combinations of surfaces, the touch frequency (36.5 times per hour) and duration per touch (14.5 s) of the face alone were the highest. Touching the hair and face at the same time had the shortest duration, at 6.7 s per touch. Note that there were no observations of triple-surface touch combination.

**Figure 2:**
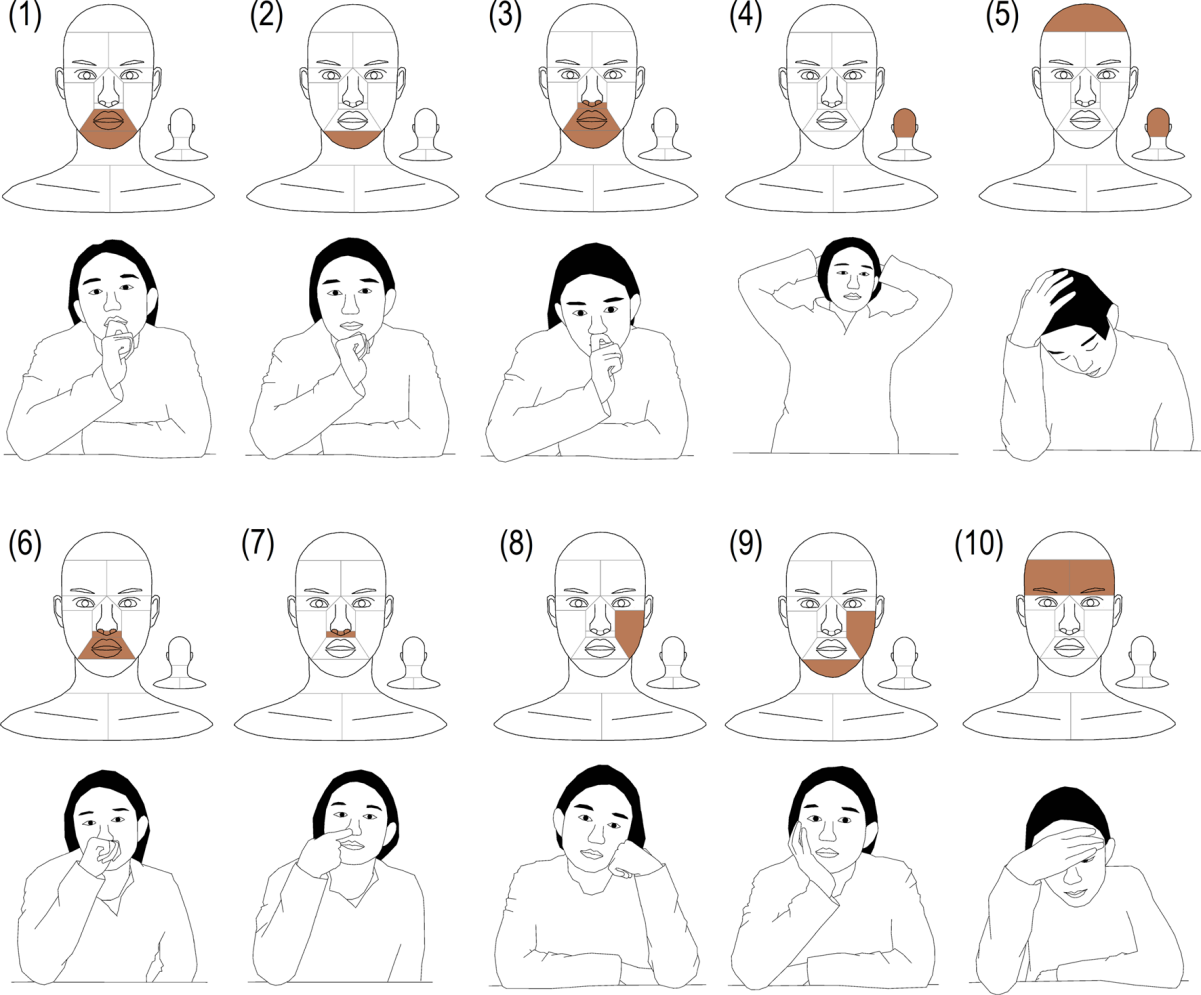
Top 10 high-frequency touch combinations. (First and third rows show the surfaces contaminated, and second and fourth rows show the possible posture of the combination.)

Due to the large area of a human hand, people usually touch more than one surface at the same time. Figure 2 lists the top ten high-frequency combinations, which account for 57.4% of the touch time. It is worth noting that four out of ten high-frequency touch combinations involved the mucous membranes. Table 4 lists detailed information about touch frequency, duration and distribution by hand.

**Table 4:** Detailed touch information about top 10 high-frequency touch combinations (value shows the mean value ± standard deviation).

No.^a^	Combination sub-surfaces	Frequency (time/h)	Duration per touch (s)	Nondominant hand percentage^b^ (%)
(1)	${F}_{M3} \& {F}_{M4}$	6.3 ± 5.0	12.0 ± 10.0	74.8 ± 25.6
(2)	${F}_{M4}$	5.4 ± 5.0	11.5 ± 8.5	77.3 ± 26.5
(3)	${F}_{M2} \& {F}_{M3} \& {F}_{M4}$	5.0 ± 6.3	12.4 ± 12.5	81.2 ± 28.8
(4)	${H}_{2}$	4.7 ± 13.2	5.4 ± 8.1	61.9 ± 30.5
(5)	${H}_{1} \& {H}_{2}$	3.4 ± 6.3	8.4 ± 7.1	63.9 ± 30.1
(6)	${F}_{M2} \& {F}_{M3}$	3.0 ± 3.7	8.6 ± 5.8	74.0 ± 30.3
(7)	${F}_{M2}$	3.0 ± 3.8	5.9 ± 6.8	75.7 ± 32.9
(8)	${F}_{L4}$	2.8 ± 3.0	10.4 ± 11.7	93.6 ± 13.7
(9)	${F}_{L4} \& {F}_{M4}$	2.0 ± 2.7	21.1 ± 23.3	89.0 ± 14.2
(10)	${F}_{L1} \& {F}_{R1}$	1.6 ± 1.9	9.3 ± 11.8	61.6 ± 36.6

^a^Combination number is the same with the sequence in Fig. 2.

^b^Percentage of touches by nondominant hand.

**Figure 3:**
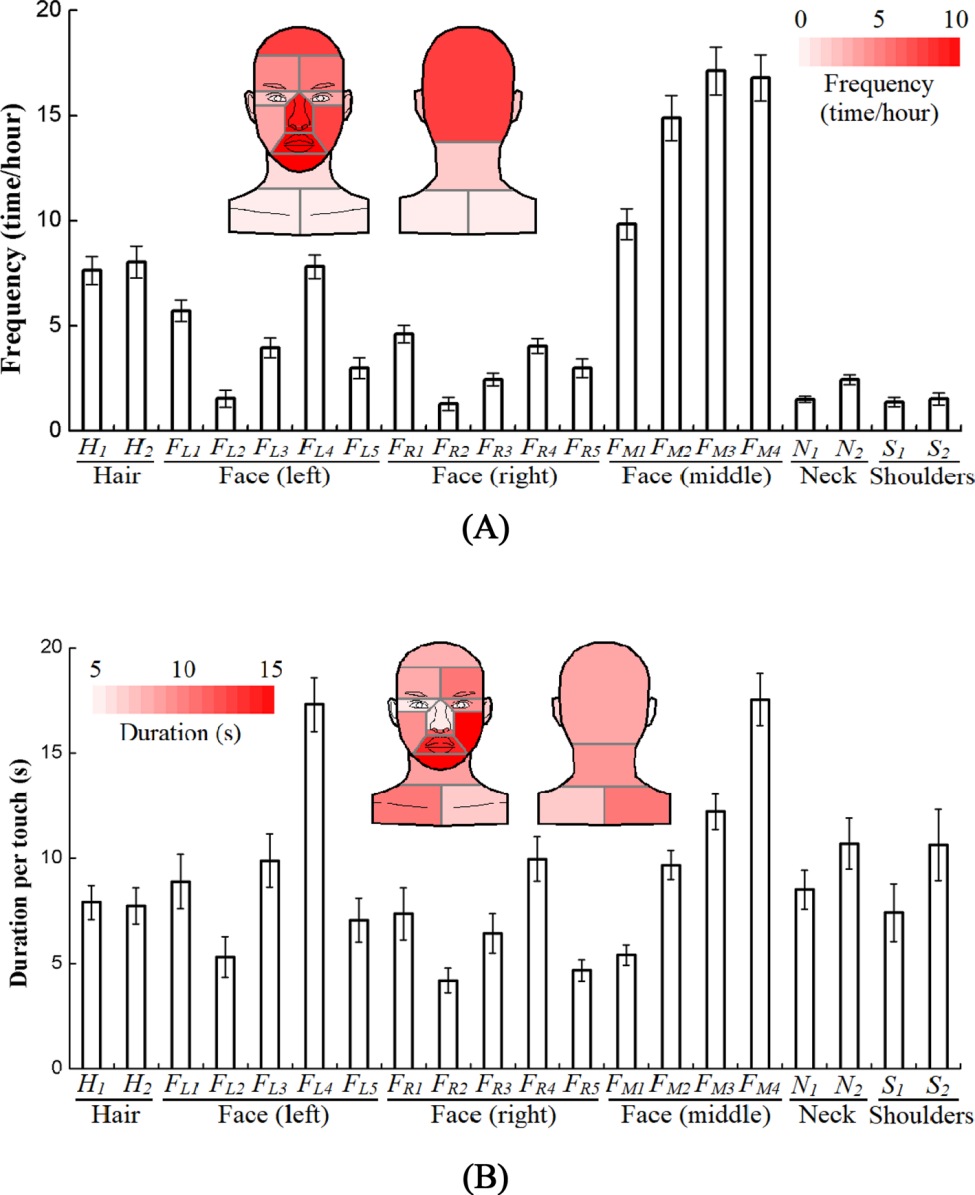
Self-touch behaviour on the 20 sub-surfaces of hair, face, neck and shoulders (means ± standard error): (**A**) touch frequency; (**B**) duration per touch. (Detailed data on human touch behaviours can be obtained in Table S1).

Figure 3 shows the touch frequency and duration per touch of the 20 sub-surfaces. All sub-surfaces on the left face had a higher probability of being touched than those on the right. Contaminating mucous membranes play an important role in the transmission of some infectious diseases, and the total touch frequency of the mucous membranes was 34.8 times/h with the standard error of 2.4 times/h. All of the sub-surfaces on the left side of the face had a higher duration per touch than those on the right. Nearly half of the incidences of face touching (36.3%) involved at least one mucous membrane contact. On the middle facial part, the lower the position of the sub-surface, the greater the duration per touch (${t}_{d-chin}>{t}_{d-mouth}>{t}_{d-nostril}>{t}_{d-nose}$), where ${t}_{d-x}$ indicates the duration per touch of surface *x*.

**Figure 4:**
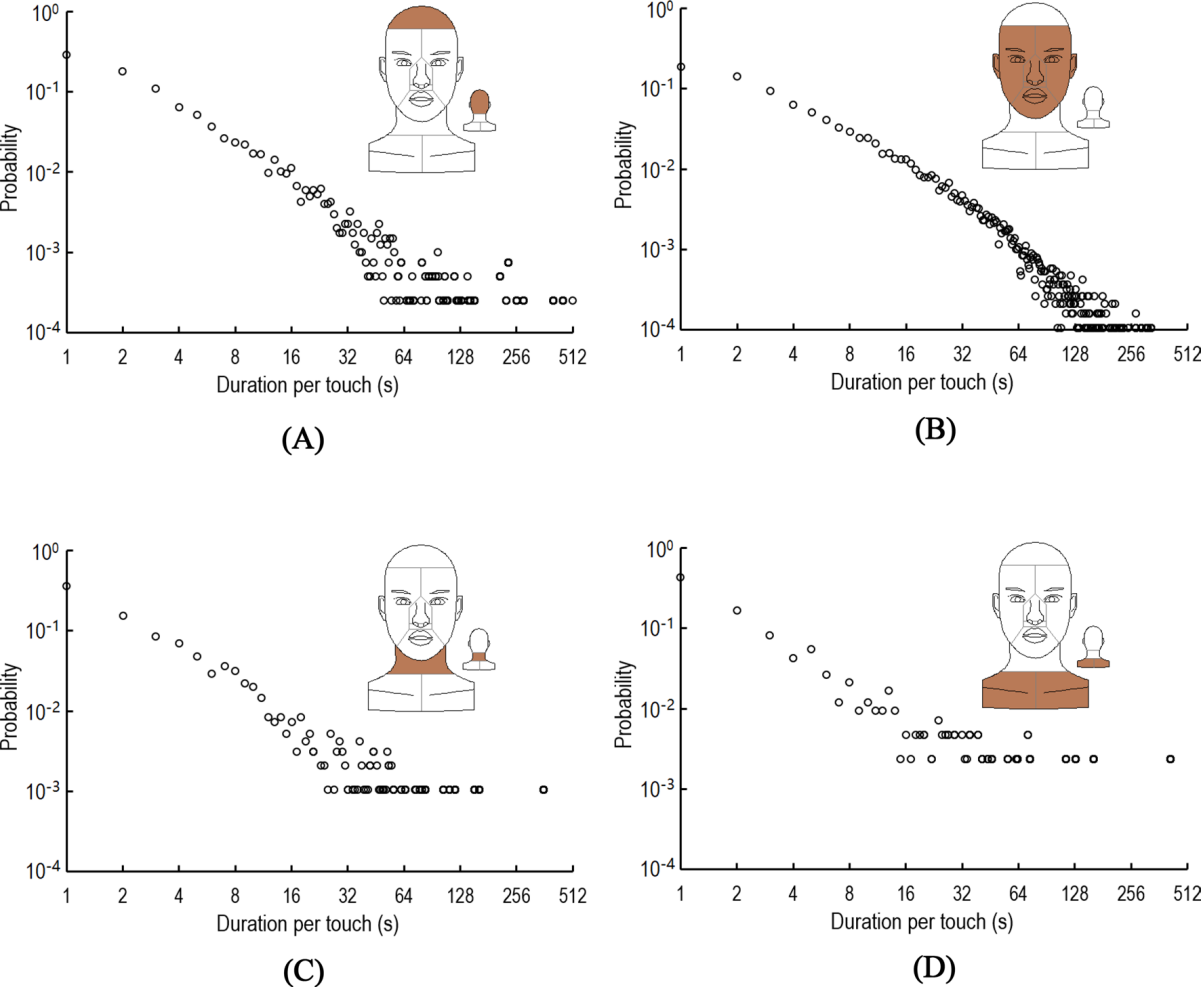
Probability distribution of duration per touch on four surfaces: (**A**) hair; (**B**) face; (**C**) neck; (**D**) shoulders.

Figure 4 shows that the probability of duration per touch on the four surfaces is in accord with the log–log linear distribution. Regardless of the surface, quick touches of less than 3 s dominated. These quick touches constituted 57.6%, 42.2%, 59.6% and 67.4% of the touches of the hair, face, neck and shoulders, respectively. Only 1.4%, 4.6%, 1.4% and 2.1% of the touches lasted longer than 1 min on the hair, face, neck and shoulders, respectively. For all surfaces, fewer than 0.2% of the touches lasted longer than 5 min.

### Touch frequency

**Figure 5:**
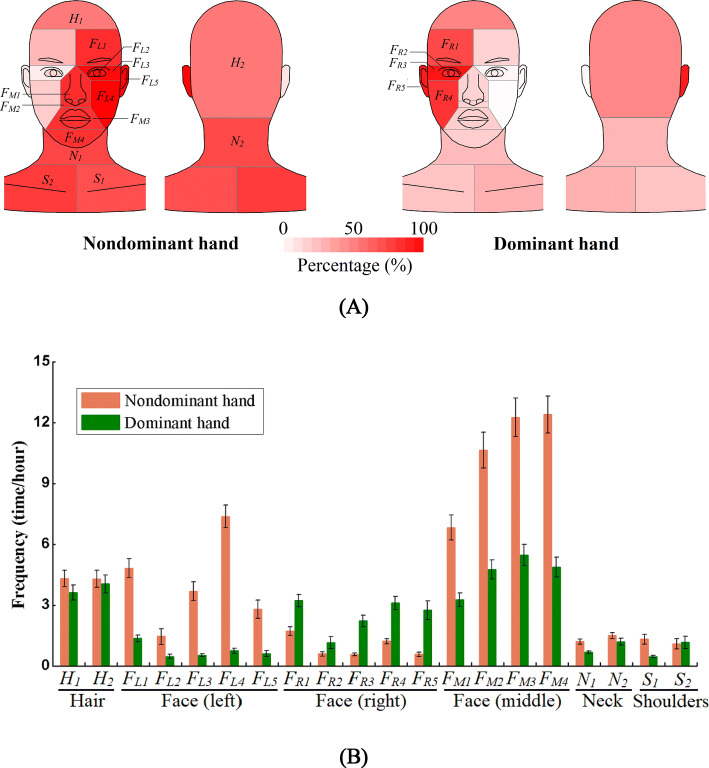
Self-touch behaviour on the 20 sub-surfaces by hand. (**A**) Percentage distribution (colour shows the percentage of touches of each sub-surface by the non-dominant and dominant hands, respectively); (**B**) touch frequency (means ± standard error) (Table S4 for the detailed data).

The 3-way ANOVA analysis of touch frequency data based on 20 sub-surfaces revealed a significant interaction between hand and sub-surface (F(19,2,960) = 16.292, P < 0.001), main effect of hand (F(1,2,960) = 87.545, P < 0.001) and sub-surface(F(19,2,960) = 47.662, P < 0.001). However, we did not observe significant main effect of gender (F(1,2,960) = 0.799, P = 0.371), interaction between gender and hand (F(1,2,960) = 0.179, P = 0.673), or a 3-way interaction of gender, hand and sub-surface (F(19,2,960) = 0.705, P = 0.817). A full description of individual and multiple interaction effects are shown in Table S2. These results indicate hand and sub-surface impact self-touch frequency (Fig. 5). Post-hoc hand comparisons within each sub-surface reveal that the nondominant hand touched all sub-surfaces on the face (except eyes) more often, whilst no significant hand differences were observed for sub-surfaces on the hair, neck and shoulder (Table S3).

There are many significant differences found in the post-hoc sub-surface comparisons within each hand: the nondominant hand shows a higher touch frequency on sub-surfaces on the left face (*F*_*L1*_, *F*_*L3*_, *F*_*L4*_) than the right face (*F*_*R1*_, *F*_*R3*_, *F*_*R4*_), but no significant differences between both eyes (*F*_*L2*_ and *F*_*R2*_), ears (*F*_*R5*_ and *F*_*R5*_), shoulders (*S*_*1*_ and *S*_*2*_), front and back hair (*H*_*1*_ and *H*_*2*_), front and back neck (*N*_*1*_ and *N*_*2*_), and among the nostril (*F*_*M2*_), lips (*F*_*M3*_) and chin (*F*_*M4*_). Both hands show the highest touch frequency on sub-surfaces on the middle of face and the lowest touch frequency on eyes (Fig. 5B). The dominant hand shows no significant differences among most sub-surfaces except the nostril (*F*_*M2*_), lips (*F*_*M3*_), chin (*F*_*M4*_) and back hair (*H*_*2*_).

**Figure 6:**
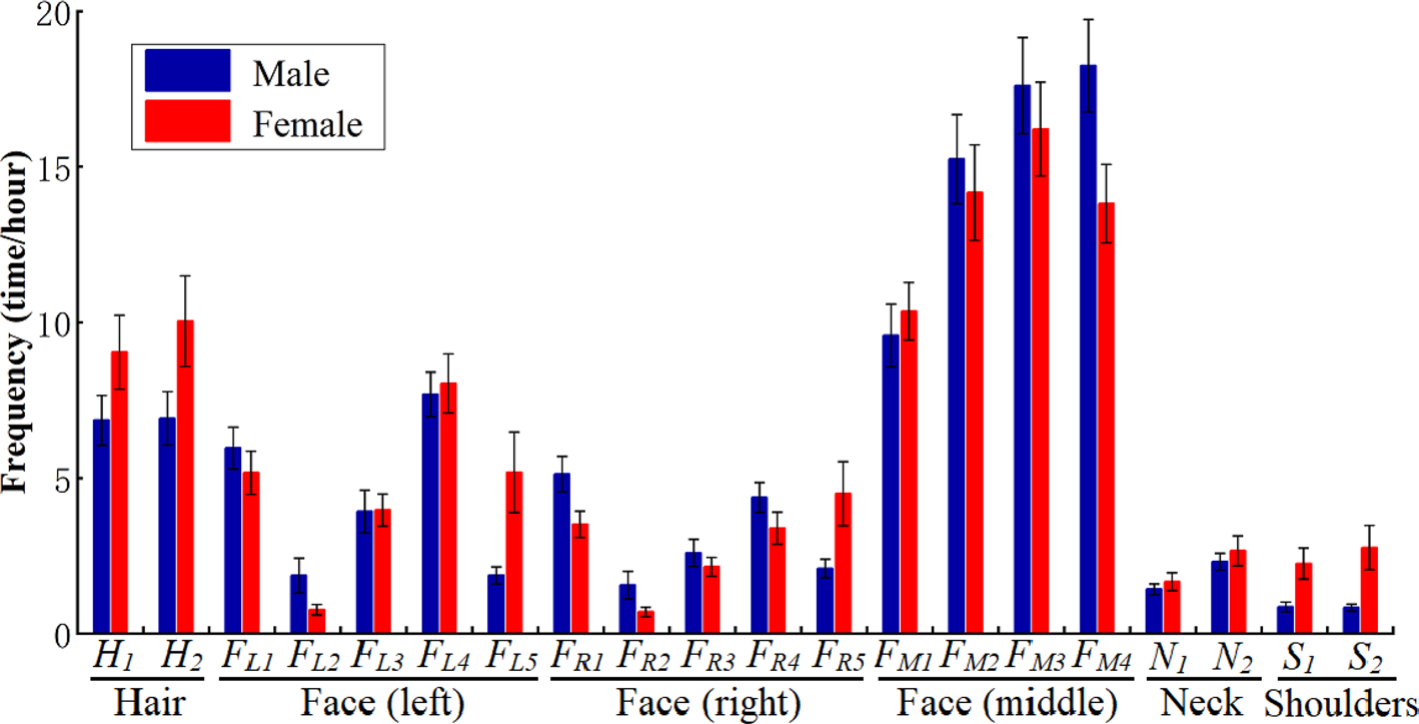
Touch frequency (means ± standard error on sub-surfaces by gender (Table S4 for the detailed data).

We observed no significant effect of gender in the touch frequency, whilst post-hoc gender comparisons within each sub-surface indicated that gender has a significant effect on touch frequency on both ears (*F*_*L5* and_ *F*_*R5*_), chin (*F*_*M4*_), and back hair (*H*_*2*_) (Table S5). Female students touched the right ear (*F*_*R5*_), left ear (*F*_*L5*_), and back hair (*H*_*2*_) more often than male students, 1.1, 1.8, and 0.4 times, respectively, whilst the male students touched the chin (*F*_*M4*_) 0.3 times more often than the female students (Fig. 6).

Table S6 shows the similar results using grouping surfaces. These results revealed hand and grouping surface impact touch frequency (Fig. S1). From post-hoc comparisons, the nondominant hand had a higher touch frequency on mucous membranes and non-mucosal face than the dominant hand, and no significant differences were observed on the hair, neck and shoulders (Tables S7 and S8). There are significant differences on touch frequency among five grouping surfaces were observed for both hands, except between the neck and shoulder in both hands, between the hair and mucous membranes, and between the hair and shoulder in the dominant hand (Tables S7 and S8).

Based on the previous research^14^, Table 5 lists the touch frequency on various body sites and surfaces in the external environment. The dominant hand touches more on personal belongings, computer, and public surfaces, while nondominant hand touches more on body and HFNS.

**Table 5:** Touch frequency on different body sites and surfaces in external environment.

Surface	Percentage by dominant hand (%)
Personal belongings (e.g. bag, cup, mobile phone)	55.0
Computer (mouse)	99.8
Computer (keyboard)	58.2
Desk	48.0
Chair	48.8
Public surface (e.g. water dispenser button, printer screen)	56.4
Body (except for HFNS)	42.6
HFNS	33.9

### Touch duration

**Figure 7:**
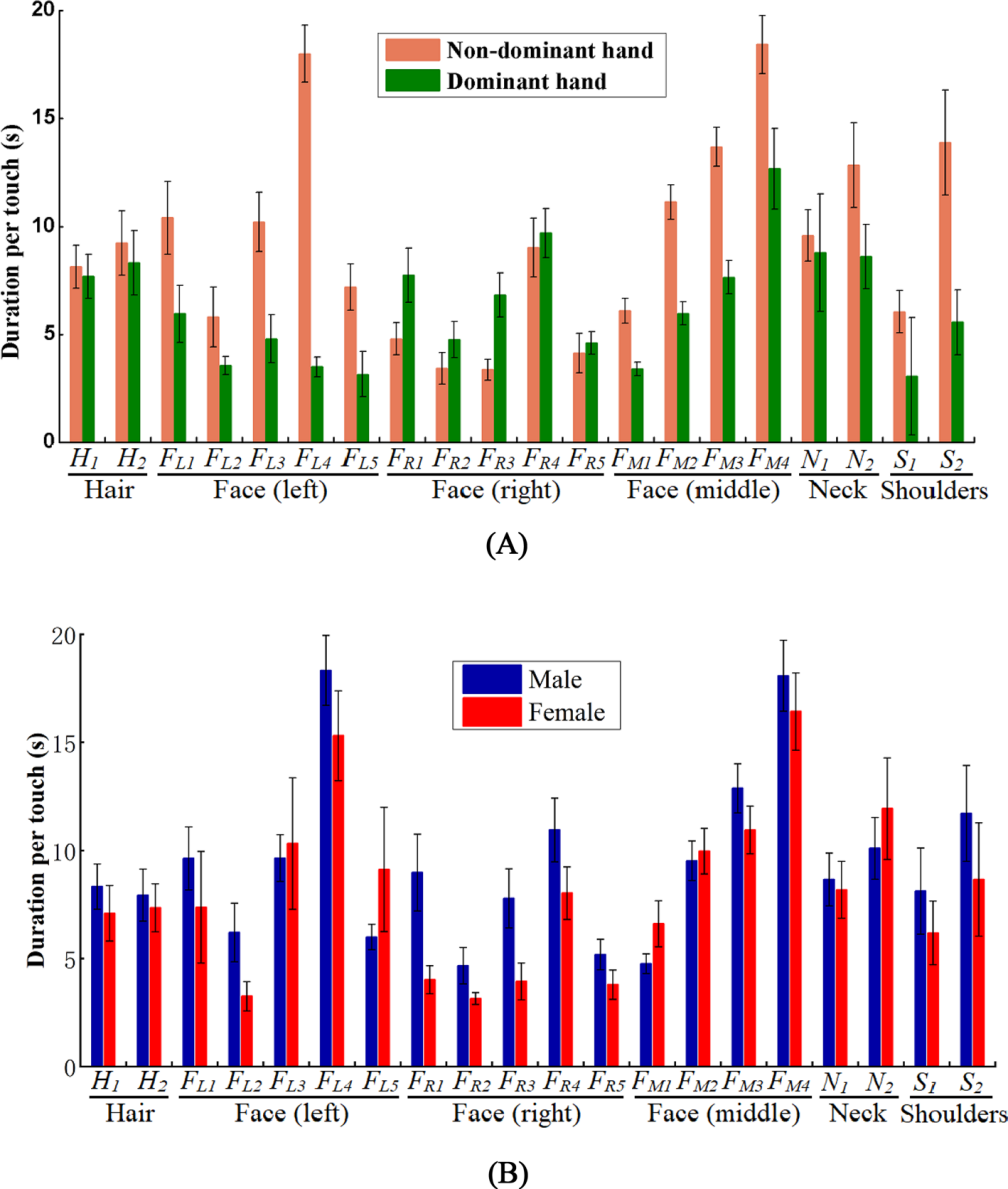
Duration per touch (means ± standard error) of 20 sub-surfaces of hair, face, neck and shoulders: (**A**) by hand; (**B**) by gender (Table S4 for detailed data).

The 3-way ANOVA analysis of duration per touch data based on 20 sub-surfaces revealed a significant interaction between hand and sub-surface (F(19,2,961) = 4.400, P < 0.001) and between gender and hand (F(1,2,961) = 6.547, P = 0.011), main effect of gender (F(1,2,961) = 6.870, P = 0.009), hand (F(1,2,961) = 39.557, P < 0.001), and sub-surface(F(19,2,961) = 7.921, P < 0.001). However, we did not observe significant interaction between gender and sub-surface (F(19,2,961) = 0.579, P = 0.924), or a 3-way interaction of gender, hand and sub-surface (F(19,2,961) = 0.589, P = 0.917). A full description of individual and multiple interaction effects can be seen in Table S2. These results indicated that gender, hand, and sub-surface impact duration per touch in the self-touch behaviour (Fig. 7). Post-hoc hand comparisons within each sub-surface reveal that the nondominant hand touched the left forehead (*F*_*L1*_), peripheric area of left eye (*F*_*L3*_), left cheek (*F*_*L4*_), nostril (*F*_*M2*_), lips (*F*_*M3*_), chin (*F*_*M4*_), back neck (*N*_*2*_) and back shoulder (*S*_*2*_) per time longer than the dominant hand, whilst no significant hand differences were observed for duration per touch on other sub-surfaces (Table S3). There are significant differences among 20 sub-surfaces for duration per touch by both hands. Figure 7A showed that the nondominant hand touched the left face longer per time than the right face, whilst the dominant hand touched the right face longer per time than the left face.

The men touched the HFNS for an average duration of 10.2 s, whilst the women, on average, touched the HFNS for 8.7 s. For the female students, duration per touch by non-dominant hand and dominant hand were 11.2 and 6.0 s, respectively; and those by the male students were 11.5 and 8.8 s, respectively (Table S9). From post-hoc comparisons, the male students touched 2.8 s longer than the female students per time for the dominant hand, whist no significant difference was observed for the nondominant hand (Table S10). There was a significant difference on duration per touch between the nondominant and dominant hand regardless of the male and female students (Table S10).

Table S6 shows similar results using grouping surfaces. Gender, hand and grouping surface impact duration per touch (Fig. S2). From Post-hoc comparisons, the nondominant hand touched non-mucosal face and mucous membranes 5.9 s longer per time than the dominant hand, whilst no significant duration differences per touch were observed on the hair, neck and shoulders (Table S11). There were significant differences between the non-mucosal face and hair, and between the mucous membranes and hair (4.9, 4.1 s, respectively) for duration per touch by the nondominant hand, whist no significant differences were observed among other grouping surfaces for duration per touch by both hands (Table S11).

### Probability matrix of surface touch

**Figure 8:**
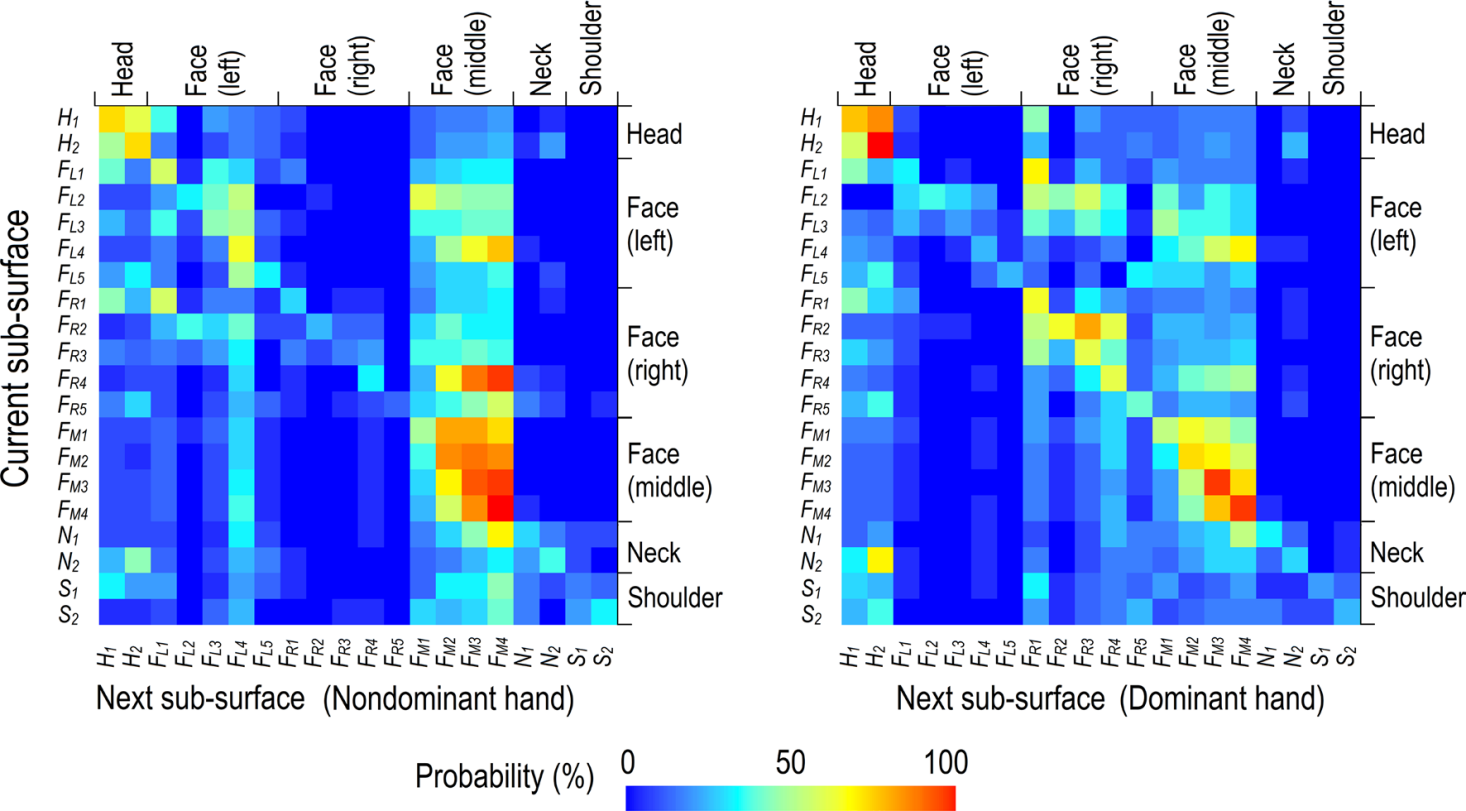
Touch behaviour matrix for probability of sequential touch on HFNS by hand (note that horizontal axis [i.e., next sub-surface] is the surface to be touched after the current sub-surface). (Actual underlying values are listed in the Tables S12 and S13).

Figure 8 shows the sequential touch matrix on the HFNS. For example, the colour of the grid (*F*_*L4*_, *F*_*M4*_) indicates the probability that *F*_*M4*_ (next sub-surface) will be touched after touching *F*_*L4*_ (the previous sub-surface). The deep red colour of most diagonal elements reveals that students had a relatively high probability of touching the same sub-surface again. The students frequently touched the middle part of their faces, as grids (*F*_*M1*_–*F*_*M4*_, *F*_*M2*_–*F*_*M4*_) are red. After touching their left/right cheeks, the students also had a relatively high probability of touching the middle part of their faces, especially their lips or chin. The hair and neck also showed a higher association. The touch frequency on the right part of the face, the neck and the shoulder was longer than that on other parts.

## Discussion

In this study, we provide a unique dataset of touches of their own HFNS by students in a Chinese postgraduate student office. This may be the first dataset to contain self-touch behaviour on all 20 regions (sub-surfaces) of the HFNS, and the data may be useful in various future studies to build models of infection transmission via the fomite route in these types of environments^21,22^.

### More self-touches of HFNS by nondominant hand and implications for microbial transfer

The dominant and nondominant hands play different roles in daily life. On average, 63.5% of the touches of surfaces in the external environment (e.g., personal belongings, chair, desk, computer and public surfaces) were made by the dominant hand. We were interested and fascinated to find that our nondominant hand is more frequently used for other private functions, such as eye-rubbing and nose picking. The nondominant hand touched the left face and middle face more often than the dominant hand significantly, while the dominant hand touched the right face more often than the nondominant hand significantly.

The average duration per touch was 13.0 s by the nondominant hand and only 8.0 s by the dominant hand, and the significant difference just occurred on the left face, middle face, back neck and right shoulder. Simultaneously, we found that the male students used the dominant hand to touch the HFNS longer than the female students per time significantly. Moreover, we found lateralisation was common, i.e. if a person touches the right side of their face, they most likely have used their right hand to do so. And this is also shown by newborns^23^.

It is known that hand influences touch behaviour, and the different roles of the two hands contribute to the dissimilarity in the skin microbiome of both hands^24^. In a microbiome analysis of dominant and nondominant hands, Fierer et al.^6^ suggested that the dominant hand contacts more types of environmental surfaces than the nondominant hand, which may explain the differences between the microbiome composition of the dominant and nondominant hands. We also found that the dominant hand touches environmental surfaces 41 times more (171 time/h versus 130 time/h) per hour than nondominant hands^14^. In addition, in this study, the most interesting finding was perhaps that 66.1% of HFNS self-touches were made by the nondominant hand. This percentage is higher than for other body sites. Fierer et al.^25^ showed that the skin microbiome can be used for forensic identification. On this basis, the microbiome on the HFNS shows greater similarity to that of the nondominant hand than to that of the dominant hand.

Staudinger et al.^26^ found that *Actinobacteria* is the most abundant phylum on the forehead, and *Propionibacterium acnes* accounts for only 72.54% of all clones. No other part of the skin has been found to be dominated by a single bacteria species^27^. The population of *Propionibacterium acnes* on the forehead can be seen as a stable individual characteristic^27,28^. Fierer et al.^6^ found that nondominant hands harbour more Proprionibacteria (35.4%) than dominant hands (30.0%). Because the two hands have similar microenvironments (e.g., humidity, serum production), this difference is most likely due to the greater frequency of facial skin touch by the nondominant hand. Leung et al.^29^ showed that the microbiome on the forehead does not have a closer relationship with the microbiome on the nondominant hand than with that on the dominant hand, but no detailed data were given for the left and right forehead. We also found that although the difference in touch frequency on the left and right forehead by two hands is evident, there is no evident difference for the whole forehead (left hand: 6.5 time/h; right hand: 4.6 time/h). *Prevotella* is one of the most common bacterial phyla on the human cheeks^30^. The touch frequency on the cheeks by the nondominant hand is 2.4 times as high as that by the dominant hand. We were interested to find that the abundance of *Prevotella* on the nondominant hand is roughly twice that on the dominant hand^6^. A concurrent analysis of bacterial communities demonstrated that physiological attributes and skin topography are two critical factors that lead to difference in microbial communities^31^. Does different touch behaviour by hand increase or decrease this difference?

Among all touches to the HFNS, 99.3% were to the participants’ own surfaces, and only 0.7% were to others’ HFNS. This may explain why the microbiomes on the dominant and nondominant hands of the same person show greater similarity to each other than to the corresponding hand of another individual^32^. Interpersonal hand microbiome variation is greater than temporal variation^33–35^. Moreover, interpersonal variation of the hand microbiome is less than the variation between body sites on the same individual^33,34^. Therefore, self-touch behaviour on the HFNS can play a very important role in influencing the microbiome distribution on different skin sites. Our HFNS are more private surfaces and are not commonly accessed by others in terms of touching. A low probability of touching other people’s skin may lead to the phenomenon that the ‘individuals with more variable hand bacterial communities have a greater variability at other skin sites, indicating microorganisms may be transferring between hand and other skin sites’^36^.

### Self-touch behaviour of mucous membranes

A high frequency of touches of the mucous membranes can lead to a high infection risk^37^. Traditionally, very few data have been available regarding self-touching behaviours on the mucous membranes of the eyes, nose and mouth (known as the T-zone)^38^. Elder et al.^38^ found that clinicians and staff in a family medicine office touched their T-zones an average of 9.5 times per hour. Hendley et al.^39^ found that the mucous membrane touch frequency with the subjects’ own hands was no more than one time per hour in medical conferences and a Sunday school. Nicas and Best^40^ found that the mean touch frequency of finger contacts with the eyes, nostrils and lips were 2.5, 5.3 and 8.0 times per hour, respectively. These earlier data were based on observational study and may carry large variabilities. We found that students touched their mucous membranes 34.9 times/h (eyes: 2.8 time/h; nostrils: 14.9 time/hour; lips: 17.1 times/h). Focusing on mucous membranes, the trend was the same as in Nicas and Best^40^: the lips are the most touched, then the nostrils, and the eyes are the least touched. However, we presented the highest self-touch frequency of our own mucous membranes among all reported data. This may be due to our use of video, which allows the full observation data set to be captured so no quick touches were missed. We also found that touch frequency on the lips and nostrils by the nondominant hand was 2.3 times as many as it by the dominant hand, and 1.9 times for duration per touch.

Our new data have significant implications for disease transmission in at least two aspects. First, although some respiratory viruses such as influenza A may have a very high inactivation rate on hands, the fomite route may still be important. For example, the influenza virus may survive for only 10 min or less on our hands, but we may touch our own mucous membranes every 2 min (assuming 34.9 times/h of self-touches of our membranes). The high frequency of touching the mucous membranes may be one reason for the similarity of bacteria distribution between the nose and the skin^10^. Thus, our data do not support the common belief that the fomite route is unimportant for influenza transmission^16–18^. Second, our face can be contaminated by large droplets when we are in close contact with an infected individual^41^. Large droplets can be deposited on our mucous membranes as in the traditional large droplet route^42^, and they can also be deposited on other parts of our HFNS. Our nondominant hand switches between touching our mucous membranes and other parts of the HFNS, which can transmit microbes between them.

### Prevention strategies for infectious disease transmission

Behaviour intervention is helpful. It is known that reducing the frequency of mucous membrane touching or face touching can potentially reduce the risk of many respiratory infections^43^. Our study showed that the touch frequency of the mucous membranes by the nondominant hand was 2.4 times that of the dominant hand (24.0 vs 9.8 times/h). Therefore, in daily life, we may reinforce the differences in our hand behaviour, for example, only using our dominant hand to touch some potential contaminated surfaces, such as flushing the toilet, water dispenser buttons and door handles. In addition, because 66.1% of the self-touches of the HFNS were by the nondominant hand and 63.5% of the touches of surfaces in the external environment were by the dominant hand, the microbiome on the nondominant hand could be swabbed to assess personal health, and that on the dominant hand could be swabbed to assess environmental threats to our health.

Researchers and organisations, including the WHO, have shown that hand hygiene is useful in reducing many respiratory and enteric infections, suggesting the importance of the fomite route^44,45^. A quantitative review found that handwashing can also reduce the risk of respiratory infection by 16%^46^. From our study, washing the nondominant hand is more important because of the higher frequency of touching the mucous membranes. In addition, not only the airborne and large droplet routes, but also the fomite route, can be effectively reduced by wearing surgical masks. The eyes, nostrils and lips are three mucous membranes on the face. Only 6.6% of touches of the mucous membranes were on the eyes, which means 93.4% of viral transfer could be blocked by wearing a surgical mask.

The presence of the cameras might have had a psychological impact, which is called Hawthorne effect, on the students’ touch behaviours. The study was limited to a single office for a period of 5 days, mainly due to the laborious nature of analysing the surface touch data from the video. Some touches could not be identified because the view was blocked by objects or students, although this was infrequent. Human touch behaviour depends on many factors, such as environment, occupation, and total indoor population. The results of the study only show the characteristics of the graduate student office. Although various types of activity recognition software are available, it is difficult to use them to study surface touch behaviour^47^. Properly designed touch surface recognition software will enable us to study surface touch behaviour as affected by individual characteristics, occupational characteristics and an enormous range of indoor environments. In addition, future studies should consider more accurate hand division, such as determining which part of the hand (five fingers, palm and the back of the hand) touches the specific surface.

## Supplementary information

Supplementary file1
