# Supplementary material for: Most self-touches are with the nondominant hand
Source: Sci Rep. 2020 Jun 26;10:10457. doi: 10.1038/s41598-020-67521-5 (PMC7320184; doi:10.1038/s41598-020-67521-5)
Supplement: Supplementary file 1 — Supplementary file1 [file 41598_2020_67521_MOESM1_ESM.docx]

**Most self-touches are with the nondominant hand**

Nan Zhang ^a^, Wei Jia^b^, Peihua Wang ^a^, Marco-Felipe King ^c^, Pak-To Chan ^a^, Yuguo Li ^a,d^

^a^ Department of Mechanical Engineering, the University of Hong Kong, Pokfulam Road, Hong Kong, China

^b^ Zhejiang Institute of Research and Innovation, The University of Hong Kong, Lin An, Zhejiang, P.R. China

^c^ School of Civil Engineering, the University of Leeds, Leeds, UK

^d^ School of Public Health, the University of Hong Kong, 7 Sassoon Road, Pokfulam, Hong Kong, China

**Supplementary Information**


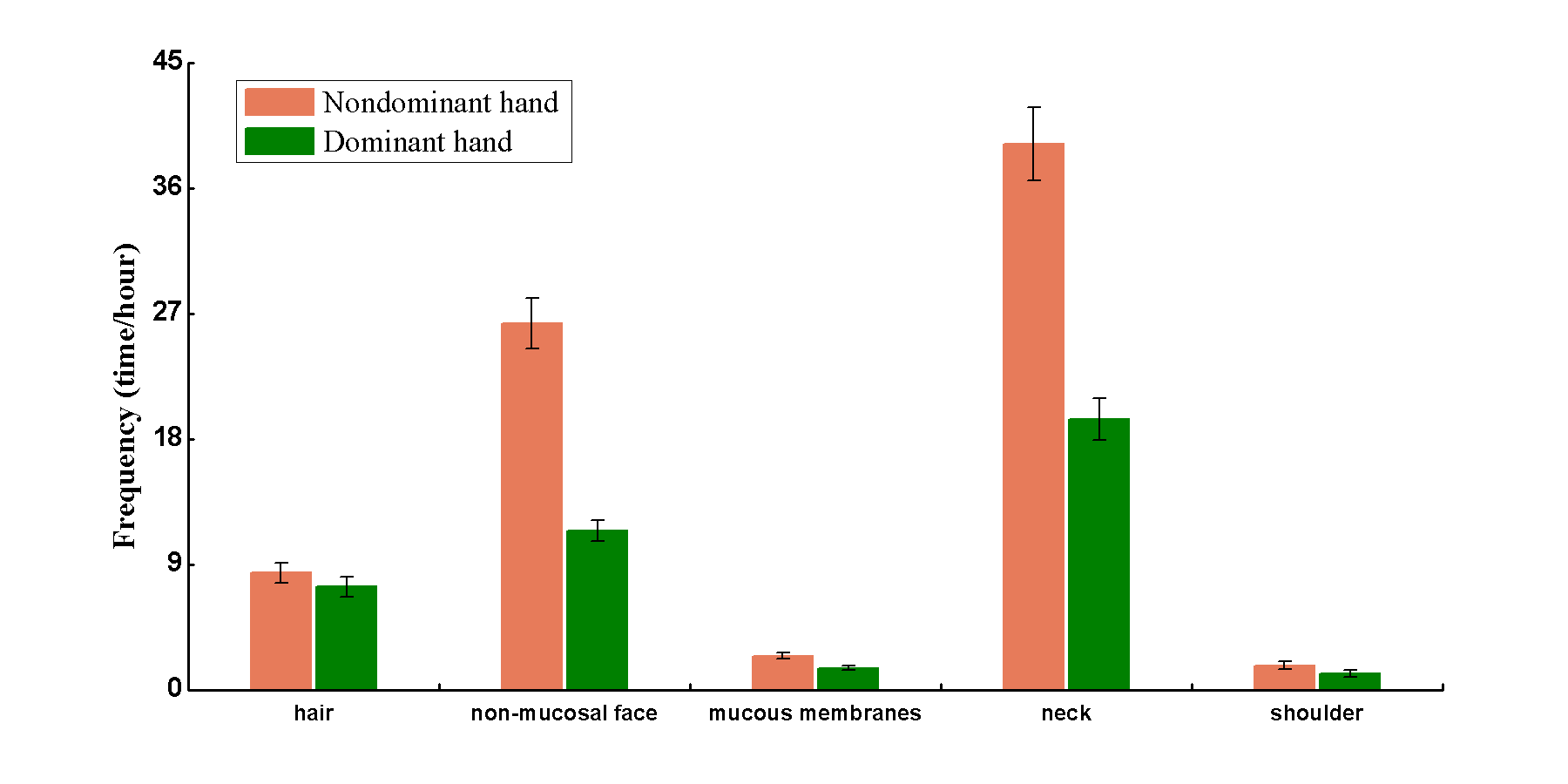


**Figure S1.** Touch frequency (means ± standard error) on 5 grouping surfaces by hand (Table S14 for detailed data)


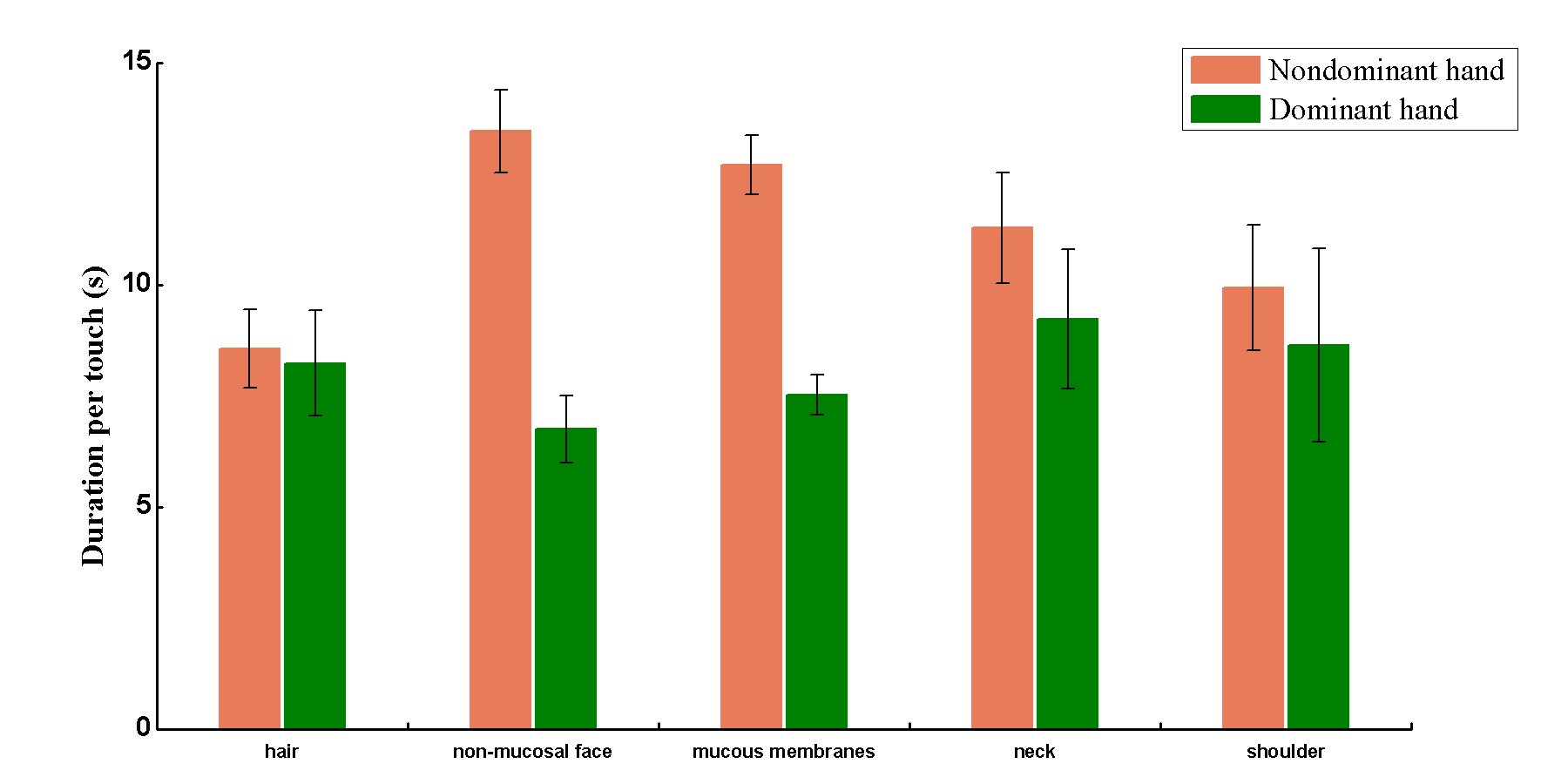


**Figure S2.** Duration per touch of 5 grouping surfaces by hand, see Table S14 for the detailed data

**Table S1.** Estimated marginal means of touch frequency and duration per touch.

| Sub- Surface | Touch frequency (time/hour) | | | Duration per touch (s) | |
| --- | --- | --- | --- | --- | --- |
|  | means | Standard error | means | | Standard error |
| *H_1_* | 7.629 | 0.670 | 7.902 | | 0.814 |
| *H_2_* | 8.026 | 0.764 | 7.731 | | 0.872 |
| *F_L1_* | 5.713 | 0.505 | 8.892 | | 1.293 |
| *F_L2_* | 1.537 | 0.399 | 5.312 | | 0.975 |
| *F_L3_* | 3.949 | 0.476 | 9.891 | | 1.278 |
| *F_L4_* | 7.815 | 0.565 | 17.307 | | 1.286 |
| *F_L5_* | 2.994 | 0.495 | 7.05 | | 1.046 |
| *F_R1_* | 4.604 | 0.413 | 7.362 | | 1.236 |
| *F_R2_* | 1.288 | 0.306 | 4.184 | | 0.581 |
| *F_R3_* | 2.439 | 0.301 | 6.425 | | 0.948 |
| *F_R4_* | 4.045 | 0.364 | 9.961 | | 1.061 |
| *F_R5_* | 2.988 | 0.438 | 4.673 | | 0.511 |
| *F_M1_* | 9.847 | 0.731 | 5.397 | | 0.476 |
| *F_M2_* | 14.886 | 1.078 | 9.678 | | 0.699 |
| *F_M3_* | 17.127 | 1.135 | 12.215 | | 0.837 |
| *F_M4_* | 16.792 | 1.092 | 17.541 | | 1.243 |
| *N_1_* | 1.508 | 0.148 | 8.510 | | 0.933 |
| *N_­2_* | 2.433 | 0.239 | 10.696 | | 1.225 |
| *S_1_* | 1.375 | 0.222 | 7.414 | | 1.37 |
| *S_2_* | 1.529 | 0.282 | 10.628 | | 1.704 |

**Table S2.** Results of touch frequency and duration per touch in terms of sub-surface in a 3-way ANOVA

| Effects | Touch frequency | | | Duration per touch | | |
| --- | --- | --- | --- | --- | --- | --- |
|  | df | F | Sig. | df | F | Sig. |
| Corrected Model | 79 | 21.083 | < 0.001 | 79 | 4.723 | < 0.001 |
| Intercept | 1 | 1193.151 | < 0.001 | 1 | 1059.027 | < 0.001 |
| gender | 1 | 0.799 | 0.371 | 1 | 6.870 | 0.009 |
| hand | 1 | 87.545 | < 0.001 | 1 | 39.557 | < 0.001 |
| sub-surface | 19 | 47.662 | < 0.001 | 19 | 7.921 | < 0.001 |
| gender * hand | 1 | 0.179 | 0.673 | 1 | 6.547 | 0.011 |
| gender * sub-surface | 19 | 1.662 | 0.036 | 19 | 0.579 | 0.924 |
| hand * sub-surface | 19 | 16.292 | < 0.001 | 19 | 4.400 | < 0.001 |
| gender * hand * sub-surface | 19 | 0.705 | 0.817 | 19 | 0.589 | 0.917 |
| Error | 2960 | -- | -- | 2961 | -- | -- |
| Total | 3040 | -- | -- | 3041 | -- | -- |
| Corrected Total | 3039 | -- | -- | 3040 | -- | -- |

**Table S3.** Univariate Tests, individual effects of hand within 20 sub-surfaces based on touch frequency and duration.

| Sub- Surface | Touch frequency | | | Duration per touch | | |
| --- | --- | --- | --- | --- | --- | --- |
|  | df | F | Sig. | df | F | Sig. |
| *H_1_* | 3000,1 | 1.196 | 0.274 | 3001,1 | 0.075 | 0.785 |
| *H_2_* | 3000,1 | 0.160 | 0.690 | 3001,1 | 0.319 | 0.572 |
| *F_L1_* | 3000,1 | 26.473 | <0.001 | 3001,1 | 6.517 | 0.011 |
| *F_L2_* | 3000,1 | 0.763 | 0.383 | 3001,1 | 0.593 | 0.441 |
| *F_L3_* | 3000,1 | 14.934 | <0.001 | 3001,1 | 6.575 | 0.010 |
| *F_L4_* | 3000,1 | 81.864 | <0.001 | 3001,1 | 58.877 | <0.001 |
| *F_L5_* | 3000,1 | 5.648 | 0.018 | 3001,1 | 2.861 | 0.091 |
| *F_R1_* | 3000,1 | 5.050 | 0.025 | 3001,1 | 2.923 | 0.087 |
| *F_R2_* | 3000,1 | 0.295 | 0.587 | 3001,1 | 0.256 | 0.613 |
| *F_R3_* | 3000,1 | 3.892 | 0.049 | 3001,1 | 2.563 | 0.109 |
| *F_R4_* | 3000,1 | 7.413 | 0.007 | 3001,1 | 0.141 | 0.707 |
| *F_R5_* | 3000,1 | 7.951 | 0.005 | 3001,1 | 0.054 | 0.815 |
| *F_M1_* | 3000,1 | 32.256 | <0.001 | 3001,1 | 2.785 | 0.095 |
| *F_M2_* | 3000,1 | 88.945 | <0.001 | 3001,1 | 10.215 | 0.001 |
| *F_M3_* | 3000,1 | 118.319 | <0.001 | 3001,1 | 14.119 | <0.001 |
| *F_M4_* | 3000,1 | 143.413 | <0.001 | 3001,1 | 12.549 | <0.001 |
| *N_1_* | 3000,1 | 0.483 | 0.487 | 3001,1 | 0.166 | 0.684 |
| *N_­2_* | 3000,1 | 0.176 | 0.675 | 3001,1 | 5.397 | 0.020 |
| *S_1_* | 3000,1 | 0.896 | 0.344 | 3001,1 | 1.941 | 0.164 |
| *S_2_* | 3000,1 | 0.008 | 0.931 | 3001,1 | 12.359 | <0.001 |

**Table S4.** Estimated marginal means of touch frequency and duration per touch on 20 sub-surfaces.

| Sub- Surface | Touch frequency (time/hour) | | | | | | | | Duration per touch (s) | | | | | | | |
| --- | --- | --- | --- | --- | --- | --- | --- | --- | --- | --- | --- | --- | --- | --- | --- | --- |
|  | Non-dominant hand | | Dominant hand | | Male | | Female | | Non-dominant hand | | Dominant hand | | Male | | Female | |
|  | means | SE | means | SE | means | SE | means | SE | means | SE | means | SE | means | SE | means | SE |
| *H_1_* | 4.322 | 0.402 | 3.626 | 0.363 | 6.866 | 0.799 | 9.046 | 1.186 | 8.131 | 0.985 | 7.682 | 1.030 | 8.334 | 1.049 | 7.097 | 1.282 |
| *H_2_* | 4.301 | 0.423 | 4.050 | 0.440 | 6.928 | 0.855 | 10.041 | 1.453 | 9.232 | 1.494 | 8.313 | 1.497 | 7.938 | 1.209 | 7.351 | 1.109 |
| *F_L1_* | 4.834 | 0.463 | 1.367 | 0.166 | 5.976 | 0.672 | 5.179 | 0.695 | 10.395 | 1.688 | 5.949 | 1.325 | 9.640 | 1.457 | 7.374 | 2.583 |
| *F_L2_* | 1.461 | 0.391 | 0.472 | 0.105 | 1.870 | 0.563 | 0.771 | 0.171 | 5.806 | 1.383 | 3.555 | 0.418 | 6.207 | 1.351 | 3.255 | 0.680 |
| *F_L3_* | 3.693 | 0.458 | 0.535 | 0.069 | 3.931 | 0.684 | 3.981 | 0.517 | 10.212 | 1.378 | 4.798 | 1.109 | 9.651 | 1.083 | 10.327 | 3.045 |
| *F_L4_* | 7.381 | 0.553 | 0.761 | 0.131 | 7.695 | 0.710 | 8.048 | 0.940 | 17.996 | 1.327 | 3.491 | 0.458 | 18.335 | 1.622 | 15.311 | 2.083 |
| *F_L5_* | 2.802 | 0.455 | 0.608 | 0.147 | 1.877 | 0.276 | 5.193 | 1.294 | 7.186 | 1.068 | 3.151 | 1.048 | 5.997 | 0.588 | 9.124 | 2.876 |
| *F_R1_* | 1.729 | 0.213 | 3.225 | 0.304 | 5.130 | 0.568 | 3.518 | 0.425 | 4.790 | 0.749 | 7.731 | 1.259 | 8.981 | 1.779 | 4.023 | 0.646 |
| *F_R2_* | 0.604 | 0.105 | 1.155 | 0.288 | 1.567 | 0.442 | 0.702 | 0.152 | 3.429 | 0.743 | 4.755 | 0.842 | 4.676 | 0.841 | 3.152 | 0.272 |
| *F_R3_* | 0.575 | 0.072 | 2.229 | 0.278 | 2.597 | 0.436 | 2.151 | 0.306 | 3.356 | 0.480 | 6.824 | 1.030 | 7.788 | 1.366 | 3.941 | 0.853 |
| *F_R4_* | 1.229 | 0.134 | 3.116 | 0.325 | 4.380 | 0.482 | 3.395 | 0.511 | 9.019 | 1.361 | 9.691 | 1.139 | 10.956 | 1.472 | 8.032 | 1.215 |
| *F_R5_* | 0.576 | 0.103 | 2.759 | 0.461 | 2.098 | 0.297 | 4.506 | 1.028 | 4.126 | 0.921 | 4.593 | 0.524 | 5.189 | 0.704 | 3.793 | 0.674 |
| *F_M1_* | 6.834 | 0.612 | 3.275 | 0.331 | 9.581 | 1.006 | 10.355 | 0.923 | 6.097 | 0.572 | 3.395 | 0.317 | 4.763 | 0.453 | 6.613 | 1.064 |
| *F_M2_* | 10.651 | 0.881 | 4.771 | 0.466 | 15.250 | 1.432 | 14.169 | 1.535 | 11.122 | 0.808 | 5.973 | 0.538 | 9.529 | 0.912 | 9.972 | 1.058 |
| *F_M3_* | 12.263 | 0.954 | 5.481 | 0.518 | 17.610 | 1.547 | 16.215 | 1.507 | 13.677 | 0.895 | 7.640 | 0.769 | 12.883 | 1.136 | 10.952 | 1.105 |
| *F_M4_* | 12.409 | 0.911 | 4.882 | 0.495 | 18.257 | 1.482 | 13.818 | 1.260 | 18.420 | 1.348 | 12.667 | 1.870 | 18.088 | 1.638 | 16.432 | 1.788 |
| *N_1_* | 1.206 | 0.128 | 0.679 | 0.068 | 1.433 | 0.173 | 1.673 | 0.285 | 9.582 | 1.194 | 8.784 | 2.720 | 8.660 | 1.225 | 8.184 | 1.318 |
| *N_­2_* | 1.502 | 0.151 | 1.205 | 0.164 | 2.323 | 0.269 | 2.667 | 0.486 | 12.841 | 1.956 | 8.602 | 1.483 | 10.105 | 1.429 | 11.939 | 2.346 |
| *S_1_* | 1.326 | 0.249 | 0.469 | 0.062 | 0.863 | 0.156 | 2.255 | 0.501 | 6.045 | 0.976 | 3.060 | 2.720 | 8.126 | 1.994 | 6.189 | 1.474 |
| *S_2_* | 1.094 | 0.249 | 1.173 | 0.304 | 0.839 | 0.112 | 2.773 | 0.707 | 13.879 | 2.430 | 5.558 | 1.516 | 11.721 | 2.221 | 8.661 | 2.618 |

SE. standard error

**Table S5.** Univariate Tests, individual effects of gender within 20 sub-surfaces based on touch frequency.

| Sub-surface | df | F | Sig. | Sub-surface | df | F | Sig. |
| --- | --- | --- | --- | --- | --- | --- | --- |
| *H_1_* | 1782,1 | 2.739 | 0.098 | *F_R4_* | 1782,1 | 0.519 | 0.471 |
| *H_2_* | 1782,1 | 5.722 | 0.017 | *F_R5_* | 1782,1 | 4.154 | 0.042 |
| *F_L1_* | 1782,1 | 0.345 | 0.557 | *F_M1_* | 1782,1 | 0.349 | 0.555 |
| *F_L2_* | 1782,1 | 0.427 | 0.514 | *F_M2_* | 1782,1 | 0.688 | 0.407 |
| *F_L3_* | 1782,1 | 0.001 | 0.971 | *F_M3_* | 1782,1 | 1.161 | 0.281 |
| *F_L4_* | 1782,1 | 0.071 | 0.790 | *F_M4_* | 1782,1 | 11.372 | 0.001 |
| *F_L5_* | 1782,1 | 5.915 | 0.015 | *N_1_* | 1782,1 | 0.029 | 0.864 |
| *F_R1_* | 1782,1 | 1.420 | 0.234 | *N_2_* | 1782,1 | 0.057 | 0.812 |
| *F_R2_* | 1782,1 | 0.257 | 0.612 | *S_1_* | 1782,1 | 0.776 | 0.378 |
| *F_R3_* | 1782,1 | 0.111 | 0.739 | *S_2_* | 1782,1 | 1.525 | 0.217 |

**Table S6.** Results of touch frequency and duration per touch in terms of 5 grouping surface in a 3-way ANOVA

| Effects | Touch frequency (time/hour) | | | Duration per touch (s) | | |
| --- | --- | --- | --- | --- | --- | --- |
|  | df | F | Sig. | df | F | Sig. |
| Corrected Model | 19 | 39.806 | < 0.001 | 19 | 3.324 | < 0.001 |
| Intercept | 1 | 620.966 | < 0.001 | 1 | 697.407 | < 0.001 |
| gender | 1 | 0.039 | 0.844 | 1 | 5.761 | 0.017 |
| hand | 1 | 58.480 | < 0.001 | 1 | 25.994 | < 0.001 |
| grouping surface | 4 | 121.367 | < 0.001 | 4 | 1.239 | 0.293 |
| gender * hand | 1 | 0.026 | 0.873 | 1 | 4.925 | 0.027 |
| gender * grouping surface | 4 | 1.261 | 0.283 | 4 | 1.029 | 0.391 |
| hand * grouping surface | 4 | 18.945 | < 0.001 | 4 | 3.150 | 0.014 |
| gender * hand * grouping surface | 4 | 0.307 | 0.873 | 4 | 0.501 | 0.735 |
| Error | 982 | -- | -- | 982 | -- | -- |
| Total | 1002 | -- | -- | 1002 | -- | -- |
| Corrected Total | 1001 | -- | -- | 1001 | -- | -- |

**Table S7.** Univariate Tests, individual effects of hand and grouping surface based on touch frequency.

| Source | df | F | Sig. |
| --- | --- | --- | --- |
| hair | 992,1 | 0.261 | 0.609 |
| non-mucosal face | 992,1 | 86.643 | <0.001 |
| mucous membranes | 992,1 | 105.807 | <0.001 |
| neck | 992,1 | 0.168 | 0.682 |
| shoulder | 992,1 | 0.058 | 0.809 |
| non-dominant hand | 992,4 | 140.011 | <0.001 |
| dominant hand | 992,4 | 26.703 | <0.001 |

**Table S8.** Pairwise comparison between hand and grouping surface based on touch frequency.

| hand | (I)grouping surface | (J)grouping surface | Mean difference (I-J) | Sig.^b^ |
| --- | --- | --- | --- | --- |
| Non-dominant hand | non-mucosal face | hair | 30.786* | < 0.001 |
|  | non-mucosal face | mucous membranes | 12.884* | < 0.001 |
|  | non-mucosal face | neck | 36.747* | < 0.001 |
|  | non-mucosal face | shoulder | 37.425* | < 0.001 |
|  | hair | mucous membranes | -17.902* | < 0.001 |
|  | hair | neck | 5.960* | 0.030 |
|  | hair | shoulder | 6.639* | 0.016 |
|  | mucous membranes | neck | 23.862* | < 0.001 |
|  | mucous membranes | shoulder | 24.541* | < 0.001 |
|  | neck | shoulder | 0.678 | 1.000 |
| Dominant hand | non-mucosal face | hair | 12.037* | < 0.001 |
|  | non-mucosal face | mucous membranes | 8.032* | < 0.001 |
|  | non-mucosal face | neck | 17.862* | < 0.001 |
|  | non-mucosal face | shoulder | 18.264* | < 0.001 |
|  | hair | mucous membranes | -4.005 | 0.259 |
|  | hair | neck | 5.825* | 0.047 |
|  | hair | shoulder | 6.228 | 0.070 |
|  | mucous membranes | neck | 9.830* | < 0.001 |
|  | mucous membranes | shoulder | 10.233* | < 0.001 |
|  | neck | shoulder | 0.402 | 1.000 |
| grouping surface | (I)hand | (J)hand | Mean difference (I-J) | Sig.^b^ |
| hair | Non-dominant hand | Dominant hand | 0.998 | 0.609 |
| non-mucosal face | Non-dominant hand | Dominant hand | 19.748* | < 0.001 |
| mucous membranes | Non-dominant hand | Dominant hand | 14.895* | < 0.001 |
| neck | Non-dominant hand | Dominant hand | 2.108 | 0.682 |
| shoulder | Non-dominant hand | Dominant hand | 2.432 | 0.809 |

Based on estimated marginal means.

*. The mean difference is significant at the .05 level.

b. Adjustment for multiple comparisons: Bonferroni.

**Table S9.** Estimated marginal means of duration per touch on hand and gender

| hand | Duration per touch (s) | | | |
| --- | --- | --- | --- | --- |
|  | Female | | Male | |
|  | means | Standard error | means | Standard error |
| Non-dominant hand | 11.228 | 0.779 | 11.477 | 0.560 |
| Dominant hand | 5.973 | 0.805 | 8.821 | 0.572 |

**Table S10.** Pairwise comparison between gender and hand based on duration per touch.

| gender | (I)hand | (J)hand | Mean difference (I-J) | Sig.^b^ |
| --- | --- | --- | --- | --- |
| Female | non-dominant hand | dominant hand | 5.255* | < 0.001 |
| Male | non-dominant hand | dominant hand | 2.656* | < 0.001 |
| hand | (I)gender | (J)gender | Mean difference (I-J) | Sig.^b^ |
| Non-dominant hand | Female | Male | -0.249 | 0.795 |
| Dominant hand | Female | Male | -2.848 | 0.004 |

Based on estimated marginal means.

*. The mean difference is significant at the .05 level.

b. Adjustment for multiple comparisons: Bonferroni.

**Table S11.** Pairwise comparison between hand and grouping surface based on duration per touch.

| hand | (I)grouping surface | (J)grouping surface | Mean difference (I-J) | Sig.^b^ |
| --- | --- | --- | --- | --- |
| Nondominant hand | non-mucosal face | hair | 4.903* | 0.007 |
|  | non-mucosal face | mucosal face | 0.766 | 1.000 |
|  | non-mucosal face | neck | 2.178 | 1.000 |
|  | non-mucosal face | shoulder | 3.528 | 1.000 |
|  | hair | mucosal face | -4.137* | 0.020 |
|  | hair | neck | -2.725 | 0.684 |
|  | hair | shoulder | -1.375 | 1.000 |
|  | mucosal face | neck | 1.412 | 1.000 |
|  | mucosal face | shoulder | 2.762 | 1.000 |
|  | neck | shoulder | 1.350 | 1.000 |
| Dominant hand | non-mucosal face | hair | -0.715 | 1.000 |
|  | non-mucosal face | mucosal face | 0.772 | 1.000 |
|  | non-mucosal face | neck | -1.710 | 1.000 |
|  | non-mucosal face | shoulder | -1.121 | 1.000 |
|  | hair | mucosal face | 1.486 | 1.000 |
|  | hair | neck | -0.995 | 1.000 |
|  | hair | shoulder | -0.406 | 1.000 |
|  | mucous membranes | neck | -2.482 | 0.809 |
|  | mucous membranes | shoulder | -1.893 | 1.000 |
|  | neck | shoulder | 0.589 | 1.000 |
| Grouping surface | (I)hand | (J)hand | Mean difference (I-J) | Sig.^b^ |
| hair | Non-dominant hand | Dominant hand | 0.318 | 0.827 |
| non-mucosal face | Non-dominant hand | Dominant hand | 5.936* | < 0.001 |
| mucous membranes | Non-dominant hand | Dominant hand | 5.942* | < 0.001 |
| neck | Non-dominant hand | Dominant hand | 2.048 | 0.192 |
| shoulder | Non-dominant hand | Dominant hand | 1.287 | 0.477 |

Based on estimated marginal means.

*. The mean difference is significant at the .05 level.

b. Adjustment for multiple comparisons: Bonferroni.

**Table S12**. Touch behaviour matrix for probability of sequential touch on HFNS by nondominant hand.

|  | *H_1_* | *H_2_* | *F_L1_* | *F_L2_* | *F_L3_* | *F_L4_* | *F_L5_* | *F_R1_* | *F_R2_* | *F_R3_* | *F_R4_* | *F_R5_* | *F_M1_* | *F_M2_* | *F_M3_* | *F_M4_* | *N_1_* | *N_­2_* | *S_1_* | *S_2_* |
| --- | --- | --- | --- | --- | --- | --- | --- | --- | --- | --- | --- | --- | --- | --- | --- | --- | --- | --- | --- | --- |
| *H_1_* | 36.12 | 30.57 | 19.31 | 0.64 | 10.22 | 8.26 | 6.14 | 5.31 | 0.00 | 0.69 | 0.69 | 0.59 | 6.68 | 9.29 | 9.78 | 10.37 | 0.74 | 3.39 | 0.93 | 0.44 |
| *H_2_* | 24.22 | 37.96 | 8.54 | 0.58 | 4.95 | 9.56 | 7.09 | 2.48 | 0.24 | 0.44 | 1.31 | 0.92 | 6.02 | 10.58 | 11.70 | 13.74 | 2.14 | 10.10 | 1.80 | 0.34 |
| *F_L1_* | 21.26 | 9.56 | 28.47 | 3.36 | 19.06 | 15.70 | 5.41 | 8.34 | 0.63 | 0.63 | 0.78 | 0.24 | 13.31 | 15.41 | 16.58 | 17.45 | 0.49 | 1.22 | 0.49 | 0.39 |
| *F_L2_* | 4.55 | 5.30 | 11.62 | 17.42 | 20.71 | 26.01 | 1.26 | 1.26 | 3.79 | 1.52 | 1.01 | 0.25 | 31.06 | 24.24 | 22.47 | 23.48 | 0.00 | 0.76 | 0.00 | 0.51 |
| *F_L3_* | 12.73 | 6.72 | 18.86 | 5.74 | 22.39 | 25.46 | 6.98 | 2.87 | 0.91 | 0.78 | 0.91 | 0.26 | 18.67 | 19.58 | 21.93 | 21.21 | 0.78 | 1.11 | 0.13 | 0.26 |
| *F_L4_* | 5.16 | 5.45 | 8.63 | 2.04 | 9.40 | 32.11 | 5.45 | 1.81 | 0.20 | 0.23 | 1.86 | 0.49 | 13.88 | 25.60 | 33.60 | 39.99 | 2.12 | 1.69 | 0.80 | 0.49 |
| *F_L5_* | 11.02 | 16.43 | 9.69 | 0.41 | 5.82 | 24.49 | 16.63 | 2.04 | 0.20 | 0.31 | 1.22 | 0.82 | 10.82 | 14.18 | 14.69 | 18.78 | 1.73 | 5.00 | 1.22 | 0.31 |
| *F_R1_* | 22.24 | 12.28 | 29.54 | 3.38 | 9.79 | 9.96 | 2.67 | 14.23 | 0.71 | 2.85 | 2.67 | 1.60 | 9.79 | 14.41 | 14.95 | 16.01 | 0.89 | 2.49 | 0.18 | 0.89 |
| *F_R2_* | 2.86 | 4.29 | 12.86 | 20.00 | 15.71 | 21.43 | 5.71 | 4.29 | 12.86 | 7.14 | 7.14 | 0.00 | 14.29 | 21.43 | 17.14 | 17.14 | 0.00 | 1.43 | 0.00 | 0.00 |
| *F_R3_* | 10.00 | 8.00 | 9.00 | 7.00 | 12.00 | 18.00 | 2.00 | 9.00 | 6.00 | 10.00 | 11.00 | 1.00 | 19.00 | 20.00 | 21.00 | 19.00 | 2.00 | 0.00 | 0.00 | 0.00 |
| *F_R4_* | 3.97 | 4.47 | 4.22 | 0.74 | 3.47 | 14.64 | 1.49 | 3.23 | 0.74 | 0.74 | 17.37 | 1.74 | 13.40 | 32.75 | 44.67 | 48.64 | 4.96 | 2.48 | 0.50 | 0.50 |
| *F_R5_* | 9.40 | 14.53 | 4.27 | 1.71 | 5.13 | 13.68 | 6.84 | 2.56 | 1.71 | 2.56 | 4.27 | 6.84 | 14.53 | 19.66 | 22.22 | 28.21 | 9.40 | 5.13 | 0.85 | 2.56 |
| *F_M1_* | 4.39 | 4.11 | 7.77 | 3.20 | 6.93 | 15.46 | 2.93 | 1.95 | 0.42 | 0.35 | 2.09 | 0.45 | 24.49 | 40.89 | 40.86 | 37.37 | 1.08 | 0.91 | 0.52 | 0.77 |
| *F_M2_* | 4.13 | 3.99 | 6.86 | 1.82 | 5.64 | 15.45 | 2.83 | 1.73 | 0.13 | 0.32 | 2.40 | 0.49 | 18.00 | 42.87 | 45.75 | 42.28 | 1.28 | 1.14 | 0.47 | 0.51 |
| *F_M3_* | 4.61 | 4.01 | 6.92 | 1.39 | 5.34 | 16.79 | 2.52 | 1.84 | 0.14 | 0.38 | 2.54 | 0.43 | 14.58 | 34.81 | 47.83 | 49.21 | 1.71 | 1.15 | 0.54 | 0.52 |
| *F_M4_* | 4.79 | 4.37 | 6.93 | 1.25 | 4.86 | 18.57 | 2.64 | 1.93 | 0.17 | 0.37 | 2.60 | 0.41 | 13.07 | 29.58 | 43.55 | 53.42 | 2.52 | 1.32 | 0.60 | 0.65 |
| *N_1_* | 4.76 | 4.98 | 4.98 | 1.08 | 3.46 | 16.45 | 5.19 | 1.52 | 0.22 | 0.43 | 2.60 | 0.65 | 8.01 | 15.37 | 22.94 | 35.28 | 15.58 | 9.96 | 4.76 | 5.41 |
| *N_­2_* | 12.04 | 22.81 | 7.48 | 0.73 | 4.56 | 12.77 | 8.21 | 2.19 | 0.00 | 0.18 | 1.82 | 1.28 | 6.39 | 9.85 | 12.23 | 17.52 | 11.68 | 18.07 | 4.01 | 0.91 |
| *S_1_* | 16.26 | 11.33 | 10.84 | 1.48 | 3.94 | 11.82 | 7.39 | 3.94 | 0.00 | 0.00 | 0.49 | 0.00 | 6.40 | 16.75 | 17.24 | 22.17 | 9.36 | 4.43 | 9.36 | 6.40 |
| *S_2_* | 3.64 | 2.42 | 5.45 | 1.82 | 7.27 | 13.33 | 1.21 | 1.82 | 1.82 | 3.64 | 2.42 | 0.61 | 15.15 | 13.33 | 15.15 | 21.82 | 9.09 | 1.82 | 10.30 | 16.97 |

**Table S13**. Touch behaviour matrix for probability of sequential touch on HFNS by dominant hand.

|  | *H_1_* | *H_2_* | *F_L1_* | *F_L2_* | *F_L3_* | *F_L4_* | *F_L5_* | *F_R1_* | *F_R2_* | *F_R3_* | *F_R4_* | *F_R5_* | *F_M1_* | *F_M2_* | *F_M3_* | *F_M4_* | *N_1_* | *N_­2_* | *S_1_* | *S_2_* |
| --- | --- | --- | --- | --- | --- | --- | --- | --- | --- | --- | --- | --- | --- | --- | --- | --- | --- | --- | --- | --- |
| *H_1_* | 39.03 | 43.07 | 5.94 | 0.44 | 0.81 | 1.03 | 0.95 | 23.11 | 1.25 | 10.12 | 7.70 | 7.41 | 7.85 | 9.68 | 9.24 | 9.83 | 0.66 | 4.84 | 0.51 | 1.54 |
| *H_2_* | 29.57 | 52.60 | 3.49 | 0.32 | 0.70 | 1.08 | 0.63 | 12.50 | 1.46 | 6.92 | 6.79 | 9.33 | 6.79 | 9.39 | 10.66 | 9.45 | 1.14 | 12.44 | 0.82 | 1.71 |
| *F_L1_* | 22.13 | 12.68 | 17.71 | 1.41 | 2.62 | 1.81 | 1.01 | 35.21 | 2.01 | 12.07 | 7.44 | 5.63 | 11.47 | 9.66 | 9.46 | 8.25 | 1.01 | 2.82 | 0.60 | 0.20 |
| *F_L2_* | 2.00 | 2.00 | 16.00 | 20.00 | 16.00 | 12.00 | 0.00 | 28.00 | 24.00 | 30.00 | 20.00 | 2.00 | 22.00 | 12.00 | 20.00 | 16.00 | 0.00 | 2.00 | 0.00 | 2.00 |
| *F_L3_* | 9.78 | 7.61 | 13.04 | 7.61 | 10.87 | 6.52 | 3.26 | 21.74 | 13.04 | 20.65 | 17.39 | 3.26 | 25.00 | 19.57 | 18.48 | 14.13 | 1.09 | 1.09 | 0.00 | 1.09 |
| *F_L4_* | 11.24 | 8.99 | 2.81 | 1.69 | 3.37 | 12.36 | 2.25 | 8.43 | 3.37 | 5.06 | 9.55 | 1.69 | 17.42 | 20.22 | 28.65 | 35.96 | 2.81 | 2.81 | 1.12 | 1.69 |
| *F_L5_* | 13.64 | 18.18 | 4.55 | 0.00 | 0.00 | 7.58 | 13.64 | 7.58 | 0.00 | 7.58 | 1.52 | 16.67 | 15.15 | 15.15 | 10.61 | 15.15 | 0.00 | 0.00 | 0.00 | 0.00 |
| *F_R1_* | 22.87 | 14.97 | 11.02 | 0.70 | 1.21 | 1.53 | 0.45 | 33.95 | 4.39 | 17.52 | 11.34 | 6.75 | 8.03 | 10.00 | 10.32 | 9.75 | 0.38 | 2.23 | 0.25 | 0.64 |
| *F_R2_* | 7.43 | 6.81 | 5.57 | 2.48 | 3.10 | 1.55 | 0.62 | 26.93 | 33.75 | 41.80 | 30.03 | 4.33 | 13.93 | 13.31 | 11.15 | 13.31 | 0.31 | 2.48 | 0.00 | 0.62 |
| *F_R3_* | 15.29 | 11.52 | 5.16 | 1.09 | 1.79 | 1.29 | 0.40 | 24.73 | 12.81 | 30.29 | 21.65 | 6.75 | 11.02 | 13.01 | 13.11 | 14.30 | 0.20 | 1.39 | 0.40 | 1.09 |
| *F_R4_* | 8.80 | 7.57 | 3.01 | 0.39 | 1.08 | 2.01 | 0.31 | 11.81 | 7.95 | 16.99 | 30.89 | 7.80 | 12.51 | 21.00 | 22.93 | 25.48 | 1.08 | 2.08 | 0.39 | 0.85 |
| *F_R5_* | 13.09 | 19.51 | 2.18 | 0.13 | 0.77 | 1.16 | 1.03 | 10.14 | 1.28 | 8.86 | 14.25 | 21.82 | 9.50 | 12.32 | 12.32 | 11.68 | 1.16 | 4.36 | 0.26 | 1.67 |
| *F_M1_* | 8.72 | 8.19 | 4.36 | 1.07 | 1.16 | 2.49 | 0.36 | 11.74 | 4.80 | 10.85 | 14.32 | 4.63 | 26.42 | 33.19 | 28.29 | 22.51 | 1.51 | 1.60 | 0.71 | 0.80 |
| *F_M2_* | 7.74 | 7.30 | 3.31 | 0.39 | 0.73 | 2.19 | 0.56 | 9.60 | 2.47 | 7.46 | 14.03 | 5.22 | 16.33 | 36.03 | 35.41 | 29.46 | 1.29 | 1.18 | 0.84 | 0.51 |
| *F_M3_* | 6.88 | 6.97 | 3.48 | 0.54 | 0.86 | 1.58 | 0.50 | 8.87 | 2.35 | 6.88 | 11.90 | 4.39 | 11.86 | 26.24 | 48.33 | 36.92 | 1.04 | 1.36 | 0.68 | 0.63 |
| *F_M4_* | 7.98 | 7.22 | 3.42 | 0.24 | 0.67 | 2.16 | 0.29 | 9.38 | 2.26 | 6.35 | 13.76 | 3.90 | 11.26 | 23.76 | 38.58 | 48.48 | 2.02 | 1.59 | 0.77 | 0.48 |
| *N_1_* | 6.06 | 10.91 | 1.21 | 0.00 | 0.61 | 2.42 | 0.61 | 4.85 | 1.82 | 4.85 | 12.73 | 9.70 | 9.09 | 12.12 | 15.76 | 27.27 | 16.36 | 7.88 | 1.82 | 3.64 |
| *N_­2_* | 17.34 | 34.42 | 2.98 | 0.00 | 0.27 | 2.17 | 0.27 | 9.76 | 1.90 | 4.61 | 8.13 | 8.40 | 7.05 | 10.30 | 14.36 | 15.72 | 7.59 | 15.45 | 0.81 | 2.98 |
| *S_1_* | 15.48 | 13.10 | 3.57 | 1.19 | 1.19 | 2.38 | 1.19 | 16.67 | 2.38 | 7.14 | 9.52 | 7.14 | 10.71 | 5.95 | 7.14 | 11.90 | 3.57 | 2.38 | 11.90 | 7.14 |
| *S_2_* | 13.43 | 18.66 | 1.49 | 0.00 | 0.00 | 1.49 | 0.00 | 11.94 | 2.99 | 7.46 | 8.21 | 12.69 | 5.97 | 7.46 | 9.70 | 8.21 | 8.21 | 5.97 | 5.97 | 13.43 |

**Table S14.** Estimated marginal means of touch frequency and duration per touch based on hand and grouping surface.

| Body | Touch frequency (time/hour) | | | | Duration per touch (s) | | | |
| --- | --- | --- | --- | --- | --- | --- | --- | --- |
|  | Non-dominant hand | | Dominant hand | | Non-dominant hand | | Dominant hand | |
|  | means | SE | means | SE | means | SE | means | Standard error |
| hair | 8.407 | 0.738 | 7.409 | 0.727 | 8.564 | 0.883 | 8.245 | 1.181 |
| non-mucosal face | 39.193 | 2.631 | 19.446 | 1.509 | 13.466 | 0.927 | 6.759 | 0.756 |
| mucous membranes | 26.309 | 1.810 | 11.414 | 0.750 | 12.701 | 0.665 | 7.531 | 0.451 |
| neck | 2.447 | 0.219 | 1.583 | 0.169 | 11.289 | 1.248 | 9.241 | 1.567 |
| shoulder | 1.769 | 0.299 | 1.181 | 0.245 | 9.938 | 1.407 | 8.652 | 2.170 |
